# Acetylcholine enhances HIF-1α signaling in pancreatic cancer cells under hypoxia through the nAChR-α7/PDPK1/YAP pathway

**DOI:** 10.7150/ijbs.117013

**Published:** 2026-01-08

**Authors:** Yunmi Cho, Ha Gyeong Kim, Ju-Hee Kang, Eun-Taex Oh, Heon Joo Park

**Affiliations:** 1 Program in Biomedical Science & Engineering, Inha University, Incheon 22212, Republic of Korea; 2 Department of Microbiology, College of Medicine, Inha University, Incheon 22212, Republic of Korea; 3 Department of Pharmacology, College of Medicine, Inha University, Incheon 22212, Republic of Korea; 4 Research Center for Controlling Intracellular Communication, College of Medicine, Inha University, Incheon 22212, Republic of Korea; 5 Department of Biomedical Sciences, College of Medicine, Inha University, Incheon 22212, Republic of Korea

**Keywords:** pancreatic cancer, acetylcholine, nAChR-α7, HIF-1α, PDPK1, YAP

## Abstract

Recent studies have extensively addressed the potential role of the autonomic nervous system, which extensively innervates the pancreas, in the development of pancreatic ductal adenocarcinoma (PDAC). Targeting hypoxia-inducible factor-1 (HIF-1) for cancer management has attracted significant research interest, in view of the finding that HIF-1 regulates the expression of various genes involved in tumor angiogenesis, metastasis, proliferation, chemoresistance, and radioresistance. In this study, we investigated the molecular mechanisms by which the neurotransmitter acetylcholine enhances the expression of HIF-1α in pancreatic cancer cells in hypoxia. Under hypoxic conditions, acetylcholine induced a concentration-dependent increase in nAChR-α7-mediated HIF-1α expression in pancreatic cancer cells* in vitro*, leading to enhanced expression of HIF-1α target genes. It also increased HIF-1α protein stability in pancreatic cancer cells under hypoxic conditions. The acetylcholine-induced elevation of HIF-1α expression was blocked by siRNA-mediated knockdown of PDPK1/YAP signaling, indicating a role for this pathway in mediating these effects. A bioinformatics analysis of publicly available clinical datasets revealed that overall survival was significantly poorer in patients with *CHRNA7* copy number amplification, whereas those with high *CHRNA7* mRNA expression showed a non-significant trend toward reduced survival, suggesting that copy number alterations have stronger clinical relevance than mRNA levels. Functionally, α-bungarotoxin, a nAChR-α7-specific inhibitor, markedly blunted the acetylcholine-induced increase in the viability of pancreatic cancer organoids under hypoxic conditions. In a mouse xenograft model, acetylcholine administration accelerated tumor growth in animals bearing control pancreatic cancer cells but not in those implanted with nAChR-α7-knockdown cells. Collectively, our findings reveal a novel mechanism of acetylcholine-induced enhancement of HIF-1α expression involving PDPK1/YAP signaling and highlight the utility of HIF-1α as a therapeutic target in acetylcholine-potentiated pancreatic cancer.

## Introduction

Recent studies have shown that the peripheral autonomic nervous system influences the growth and progression of several solid tumor types by regulating gene expression, both within the primary tumor and in its surrounding microenvironment 1-3. The autonomic nervous system is divided into sympathetic and parasympathetic branches, which exert varying effects on cancers depending on the tumor type and stage 2. Pancreatic ductal adenocarcinoma (PDAC) develops from the exocrine part of the pancreas and is associated with poor prognosis 4. Both the parasympathetic and sympathetic nervous systems densely innervate the pancreas and regulate exocrine secretions of acinar and ductal cells 2,3. Recent studies on the involvement of the autonomic nervous system in PDAC development have revealed a more complex and unexpected processes compared with other cancer models 2. For example, parasympathetic cholinergic signaling is reported to exert an antitumor effect against PDAC 5,6, whereas the sympathetic nervous system induces pancreatic tumorigenesis 7. In view of the widespread distribution of the autonomic nervous system in the pancreas and the need to improve treatment efficacy, an extensive examination of the effects of autonomic neurotransmitters on the malignant transformation of pancreatic cancer is warranted.

A number of neurotransmitters have been implicated in tumor progression 8, including the peripheral autonomic neurotransmitters epinephrine, norepinephrine, dopamine, serotonin, and acetylcholine 9. Epinephrine and norepinephrine play roles in stress-induced cancer development, including cell proliferation, survival, migration and tumor growth processes, through β-adrenergic receptor (βAR), cyclic AMP (cAMP), and protein kinase A (PKA) pathways 10,11. Dopamine exerts an antiproliferative effect on cancer cells, an action opposite that of norepinephrine 12, whereas excessive expression of serotonin in breast cancer cells serves to sustain their growth 13. The biological activity of acetylcholine, a neurotransmitter of the parasympathetic system, is mediated by nicotinic acetylcholine receptors (nAChRs) and muscarinic acetylcholine receptors (mAChRs) in both central and peripheral nervous systems 14.

Schuller and coworkers (1989) highlighted a potential role of the autonomic nervous system in cancer cell regulation 8, showing that nicotine stimulates the proliferation of human small-cell lung cancer through binding to nAChRs 15. In recent years, nAChRs have been identified as key regulators of mesothelioma cell growth 16; colon cancer cell proliferation 17; and gastric cancer cell proliferation, angiogenesis, and migration 18. mAChRs have additionally been shown to play regulatory roles in the proliferation of colon 19 and breast cancer cells 20. Nicotine is reported to act through nAChRs and hypoxia-inducible factor-1 (HIF-1) signaling to induce an increase in epithelial-to-mesenchymal transition (EMT) in PDAC 21, a pathway in which nAChR-α7 functions as the primary receptor 22-24. These findings have further fueled research aimed at establishing whether pancreatic acetylcholine induces PDAC progression through regulation of nAChR-α7-mediated HIF-1 signaling.

Hypoxia is an important feature of the microenvironment of solid tumors, particularly PDAC, owing to their avascular morphology 21. In cancer cells, hypoxia activates the HIF signaling pathway, in turn regulating the expression of several genes involved in angiogenesis, tumor growth, metabolic reprogramming, metastasis, chemoresistance, and radioresistance 25. Therefore, the HIF signaling system is considered a crucial target for cancer treatment 26. HIFs are heterodimeric transcription factors composed of one of three oxygen-regulated α-subunits, HIF-1α, HIF-2α and HIF-3α, and a constitutively expressed hydrocarbon receptor/nuclear translocator β subunit, HIF-1β 25,27-30. HIF-1 function requires the formation of a HIF-1α/HIF-1β heterodimer, in which HIF-1α serves as the major regulatory subunit responsible for the transcriptional function of the complex 31. In well-oxygenated cells, the prolyl hydroxylase/von Hippel-Lindau (PHD/pVHL) signaling pathway induces proteasome-mediated degradation of HIF-1α 32. Under hypoxic conditions, deficits in O_2_, ferrous iron and α-ketoglutarate, which serve as substrates for PHDs, inhibit proteasomal degradation of HIF-1α protein, thereby increasing its expression 25. Following hypoxia-induced stabilization, HIF-1α translocates with HIF-1β to the nucleus, where it binds to hypoxia-response elements (HREs) and induces the transcription of various genes involved in cellular adaptation and survival in hypoxic environments 25,32.

Yes-associated protein (YAP) is a transcriptional coactivator of the Hippo pathway 33. Phosphorylation of YAP at serine 397 inhibits YAP migration from the cytoplasm into the nucleus, leading to decreased activity 34. Cancer biology studies indicate that YAP is overexpressed in several malignancies and contributes to tumor formation, cancer cell proliferation, and unfavorable clinical outcomes 21,34,35. Another study showed that YAP contributes to the stabilization of nuclear HIF-1α protein by nicotine in hypoxia, in turn promoting EMT and tumor progression of pancreatic cancer cells 21. These observations highlight the need for further research to understand the mechanisms underlying acetylcholine-induced nuclear translocation of YAP and the subsequent increase in HIF-1α protein stability in pancreatic cancer cells under hypoxic conditions.

Abnormal activation of the phosphoinositide 3-kinase (PI3K) signaling pathway, which is commonly observed in cancer, leads to dysregulation of several intracellular processes that are typically controlled by PI3K, including cell survival, growth, proliferation, and migration 36. Downstream effectors of PI3Ks that play key roles in several cancer types include protein kinase B (PKB)/Akt and 3-phosphoinositide-dependent protein kinase 1 (PDPK1). Recent studies have shown that mutant forms of PDPK1 are critical components of the oncogenic PI3K signaling pathway in breast cancer, suggesting that blocking PDPK1 activity could inhibit disease progression 36. Consistent with this, hyperactivation of the PI3K/PDPK1/Akt signaling pathway is observed in numerous human cancers 36. PDPK1 is a master regulator and phylogenetically conserved member of the large AGC family of serine and threonine kinases, so-named because they exhibit sequence homology to the catalytic domain of cAMP-dependent kinase 1 (PKA), cGMP-dependent protein kinase (PKG), and protein kinase C (PKC) 36,37. PDPK1 is frequently elevated in human breast cancer compared with normal breast epithelial cells and is correlated with activity of the upstream PI3K signaling pathway 38,39. Upregulation or activation of PDPK1 has additionally been reported in acute myeloid leukemia and ovarian cancer 40,41. In pancreatic cancer, induction of PI3K signaling mediated by Kirsten Rat Sarcoma Viral Oncogene Homolog (KRAS) mutants has been reported as a key tumor-initiating event 42. According to previous findings, PI3K-induced PDPK1 activation is mediated by the Hippo pathway, and leads to translocation of YAP from the cytosol to the nucleus 36. Nicotine is reported to activate nAChR-α7-mediated PI3K signaling in lung cancer 43,44. In pancreatic cancer, nicotine activates nAChR-α7, leading to Src-mediated PI3K signaling 45. However, the mechanisms underlying nAChR-α7 activation by acetylcholine in pancreatic cancer cells in a hypoxic environment and its effects on PDPK1-induced activation of the YAP/HIF-1α signaling pathway remain unclear.

In the current study, we investigated the mechanisms by which acetylcholine enhances HIF-1α expression in pancreatic cancer cells as well as the viability of pancreatic cancer organoids under hypoxia. Our results indicate that acetylcholine enhances expression of HIF-1α in a dose-dependent manner through promoting nAChR-α7-mediated translocation of YAP from the cytosol to the nucleus. In pancreatic cancer cells under hypoxia, acetylcholine-mediated PDPK1 activation regulated the translocation of cytosolic YAP to the nucleus. Consistent with this, we found that inhibiting nAChR-α7 with the selective inhibitor, α-bungarotoxin, suppressed the acetylcholine-induced increase in the viability of pancreatic cancer organoids under hypoxic conditions.

Given that pancreatic tumors are known to be profoundly hypoxic, with oxygen partial pressures in patient-derived tissues ranging from 0 to 5.3 mmHg 46, the hypoxic conditions used in our *in vitro* models (0.5% O_2_, ~3.8 mmHg) are considered physiologically relevant. To further validate our findings *in vivo*, we employed a xenograft mouse model using pancreatic cancer cells with and without nAChR-α7 expression. Following tail vein injection of acetylcholine, tumor growth was enhanced only in mice bearing tumors derived from nAChR-α7-expressing cells, supporting the role of the cholinergic pathway in promoting pancreatic tumor progression in hypoxia.

## Materials and Methods

### Pancreatic cancer cells and cultures

The human PDAC cell lines, MIA-PaCa-2, AsPC-1, HPAF-II, Panc-1, Capan-1 and BxPC-3, were purchased from American Type Culture Collection (ATCC, USA). Cells were cultured in Dulbecco's modified Eagle's medium (DMEM), Minimum Essential medium (MEM) or RPMI-1640 medium, as recommended, at 37 °C in a humidified, 5% CO_2_ environment, unless otherwise specified. A hypoxic environment was established by culturing cells in an InvivoO_2_ 500 hypoxia workstation (The Baker Company, USA) set to maintain 0.5% O_2_, 5% CO_2_, and balanced N_2_. For normoxic controls, cells were maintained at 20% O_2_ under otherwise identical conditions.

### Chemicals and antibodies

Acetylcholine, cycloheximide (CHX), MG132, scopolamine and α-bungarotoxin were purchased from Sigma-Aldrich (USA). Lipofectamine 2000 reagent was obtained from Thermo Fisher Scientific (USA). The indicated antibodies were acquired from the following suppliers: anti-HIF-1α (1:1000, #36169; Cell Signaling Technology, USA), anti-β-actin (1:1000, #A1978; Sigma-Aldrich, USA), anti-HIF-1β (1:1000, #3414; Cell Signaling Technology), anti-AKT (1:1000, #9272; Cell Signaling Technology), anti-p-AKT (1:1000, #4060; Cell Signaling Technology), anti-YAP (1:1000, #14074; Cell Signaling Technology), anti-p-YAP (1:1000, #53749; Cell Signaling Technology), anti-p-PDK (1:1000, #3438; Cell Signaling Technology), anti-PDK (1:1000, #5662; Cell Signaling Technology), anti-histone H3 (1:1000, #4499; Cell Signaling Technology), anti-PI3K (1:1000, #4292; Cell Signaling Technology), anti-p-PI3K (1:1000, #4228; Cell Signaling Technology), anti-S6K (1:1000, #9202; Cell Signaling Technology), anti-p-S6K (1:1000, #9205; Cell Signaling Technology), and anti-nAChR-α7 (1:1000, #AB216485; Abcam, USA). HRP-conjugated anti-rabbit (1:3000, #7074; Cell Signaling Technology) and anti-mouse (#7076; Cell Signaling Technology) secondary antibodies were used as appropriate.

### RNA isolation and quantitative polymerase chain reaction

Total RNA was extracted from cancer cells using TRIzol reagent (Invitrogen, USA) and subsequently treated with DNase I (New England Biolabs, USA). cDNA was synthesized from total RNA (1 μg) using AccuPower RT PreMix (Bioneer Corporation, Daejeon, Republic of Korea), and the following targets were amplified by polymerase chain reaction (PCR) using the indicated primer pairs: *PDK1*, 5'-TTC ACG ATC TGG ACT CGA ACT-3' (forward) and 5'-TTA TCC ACT GGG CCC ATC TTT-3' (reverse); *PGK1*, 5'-ACC TTG CCT GTT GAC TTT GTC-3' (forward) and 5'-GTG ACA GCC TCA GCA TAC TTC-3' (reverse); *LDHA*, 5'-CAT CCT GGG CTA TTG GAC TCT-3' (forward) and 5'-TGT CCC AAA ATG CAA GGA ACA-3' (reverse); *SLC2A*, 5'-CGG GAT CCA TGG AGC CCA CCA GCA AGA AGC-3' (forward) and 5'-GCT CTA GAT CAC ACT TGG GAA TCA GCC C-3' (reverse); *CA9*, 5'-TCT ACC TGG AGA GGA GGA TCT-3' (forward) and 5'-GTC CCT GTG GGC ATT ATT CTG-3' (reverse); and *18S rDNA*, 5'-CTG ACC CTG CAC TCA ATC AAG-3' (forward) and 5'-TGG GAC TAT TAG GCT CAG GTG-3' (reverse) (Bioneer Corporation). Quantitative polymerase chain reaction (qPCR) analyses were performed using a CFX Connect Real-Time PCR Detection System (Bio-Rad, USA).

### RNA isolation and RT-PCR

For reverse transcription-PCR (RT-PCR), total RNA was extracted from cancer cells or tissues using TRIzol reagent (Invitrogen) and subsequently treated with DNase I (New England Biolabs). cDNA was synthesized from 1 μg total RNA using AccuPower RT PreMix (Bioneer Corporation) and the following targets were amplified by PCR using the indicated primer pairs (Bioneer Corporation): *CHRM1*, 5'-CAG GCA ACC TGC TGG TAC TC-3' (forward) and 5'-CGT GCT CGG TTC TCT GTC TC-3' (reverse);* CHRM2*, 5'-CTC CTC TAA CAA TAG CCT GG-3' (forward) and 5'-GGC TCC TTC TTG TCC TTC TT-3' (reverse);* CHRM3*, 5'-GGA CAG AGG CAG AGA CAG AA-3' (forward) and 5'-GAA GGA CAG AGG TAG AGT GG-3' (reverse); *CHRM4*, 5'-ATC GCT ATG AGA CGG TGG AA-3' (forward) and 5'-GTT GGA CAG GAA CTG GAT GA-3' (reverse); *CHRM5*, 5'-ACC ACA ATG CAA CCA CCG TC-3' (forward) and 5'-ACA GCG CAA GCA GGA TCT GA-3' (reverse); *β-actin*, 5'- CAC TCT TCC AGC CTT CCT TC-3' (forward) and 5'-CTC GTC ATA CTC CTG CTT GC-3' (reverse); *CHRNA1*, 5'-CGT CTG GTG GCA AAG CT-3' (forward) and 5'-CCG CTC TCC ATG AAG TT-3' (reverse); *CHRNA2*, 5'-CCG GTG GCT TCT GAT GA-3' (forward) and 5'-CAG ATC ATT CCA GCT AGG-3' (reverse); *CHRNA3*, 5'-CCA TGT CTC AGC TGG TG-3' and 5'-GTC CTT GAG GTT CAT GGA-3'; *CHRNA4*, 5'-GAA TGT CAC CTC CAT CCG CAT C-3' and 5'-CCG GCA ATT GTC CTT GAC CAC-3'; *CHRNA5*, 5'-TCA TGT AGA CAG GTA CTT C-3' and 5'-ATT TGC CCA TTT ATA AAT AA-3'; *CHRNA6*, 5'-GGC CTC TGG ACA AGA CAA-3' and 5'-AAG ATT TTC CTG TGT TCC C-3'; *CHRNA7*, 5'-CTG CAG CGA GTG GAA GTT CG-3' and 5'-GGA CAC GGC CTC CAC GAA GT-3'; *CHRNA9*, 5'-GTC CAG GGT CTT GTT TGT-3' and 5'-ATC CGC TCT TGC TAT GAT-3'; *CHRNA10*, 5'-CTG TTC CGT GAC CTC TTT-3' and 5'-GGA AGG CTG CTA CAT CCA-3'; *CHRNB2*, 5'-CGG CTC CCT TCC AAA CAC A-3' and 5'-GCA ATG ATG GCG TGG CTG CTG CA-3'; *CHRNB3*, 5'-AGA GGC TCT TTC TGC AGA-3' and 5'-GCC ACA TCT TCA AAG CAG-3'; *CHRNB4*, 5'-CTG AAA CAG GAA TGG ACT-3' and 5'-CCA TGT CTA TCT CCG TGT-3' (reverse). PCR products were analyzed by electrophoresis on 1.5% agarose gels and visualized by staining with ethidium bromide.

### Nuclear and cytoplasmic protein extraction

The cytoplasmic fraction was obtained by resuspending cells in 300 μL Tween-20 lysis buffer (25 mM Tris, pH 8.0, 0-50 mM NaCl, 2 mM EDTA, 1 mM phenylmethylsulfonyl fluoride [PMSF], 0.5% Tween-20). After incubating samples on ice for 15 min, cytoplasmic proteins were harvested by centrifugation at 6000 g for 1 min at 4°C and collecting the supernatant. Pellets, containing nuclear proteins, were resuspended in 100 μL RIPA buffer containing 500 mM NaCl and incubated on ice for 15 min. Nuclear proteins were harvested by centrifugation at 10,000 g for 1 min at 4°C. Samples were assessed using sodium dodecyl sulfate-polyacrylamide gel electrophoresis (SDS-PAGE) and immunoblot analyses. Immunoblot results were evaluated with the aid of chemiluminescence and ChemiDoc (Bio-Rad).

### Immunoprecipitation and Immunoblot analyses

Cells were lysed with ice-cold RIPA buffer containing a protease inhibitor cocktail (Roche Applied Science, Germany), sodium orthovanadate (Sigma-Aldrich), and sodium fluoride (Sigma-Aldrich). Proteins in whole-cell lysates were resolved using SDS-PAGE and subjected to immunoblot analysis. Signals were detected using enhanced chemiluminescence reagent (Thermo Fisher Scientific). For endogenous co-immunoprecipitation, total cell protein (1 mg) was incubated with 25 μL washed Protein G-magnetic beads (New England Biolabs) at 4°C for 1 h. Cleared lysates were incubated first with 1 μg anti-HIF-1α or normal mouse IgG antibody overnight at 4 °C, and then with 25 μL washed Protein G-magnetic beads at 4 °C for 1 h. The immunoprecipitation matrix-antibody complex was washed three times with ice-cold RIPA/PBS (33% v/v). Bound proteins were resolved by SDS-PAGE, followed by immunoblot analysis. Signals were detected using enhanced chemiluminescence (Thermo Fisher Scientific). Uncropped images of blots are presented in **Supplementary Figure S10**.

### Transfection of siRNA

Knockdown of YAP, HIF-1α or PDPK1 was achieved by RNA interference using 19-bp small interfering RNAs (siRNAs) containing a 2-deoxynucleotide overhang. Specific siRNAs targeting *YAP* (CAGAAGAUCAAAGCUACUUdtdt), *HIF-1α* (GAAUGAAGAGGCGAAGAUUdTdT) or *PDPK1* (GAGGAAUUGUGGAGACUGUdtdt), as well as non-targeting siRNA (negative control), were purchased from Bioneer Corporation. Cells were seeded in 25 cm^2^ flasks, grown to ~80% confluence, and transfected with siRNA duplexes using Lipofectamine 2000 (Invitrogen), in accordance with the manufacturer's protocol. After a 48-h period, cells were processed for analysis as indicated.

### Reporter assays

Cells (5 × 10^4^) were initially seeded in 25 cm^2^ flasks, incubated overnight, and co-transfected with a 50-μL mixture containing 1 μg p3×HRE-*luc* (Addgene, USA) plasmid, 0.01 μg pRL-*luc* (transfection control; Promega, USA) and TurboFect *in vitro* transfection reagent (Thermo Fisher Scientific). After 16 h, cells were exposed to 20% or 0.5% O_2_ for 24 h. Luciferase activity was determined using an assay system (Promega) and normalized to that of *Renilla* luciferase, following the manufacturer's instructions. Three independent transfection experiments were performed in each case.

### Construction of plasmids and stable cell lines

The plasmid shnAChR-α7 was constructed using the nucleotides, 5'-TTT AGT GAG TCT GGT TTA TCC GGT GTT TCG TCC TTT C-3' (forward) and 5'-CTC GAG TTT AGT GAG TCT GGT TTA TTT TTT GAA TTC GAT ATC ATC-3' (reverse) (Bioneer Corporation). After hybridization of nucleotides, the resulting double-stranded shnAChR-α7 DNA was cloned and directly ligated into a pshCont vector (Vector Builder, USA) for further cloning. Cloned plasmids were analyzed by DNA sequencing (Bionics, Seoul, Republic of Korea). Stable cell lines were constructed by seeding cells at a density of 5 × 10^4^ cells per well in 24-well plates and transfecting them with a 50-μL mixture containing 1 μg DNA and TurboFect *in vitro* transfection reagent (Thermo Fisher Scientific). Transfected cells were selected by culturing with 1 μg mL^-1^ puromycin (Duchefa Biochemie, Netherlands) for 7 days and were maintained in DMEM containing 0.3 μg mL^-1^ puromycin during experiments.

### Organoid culture

Patient-derived pancreatic cancer organoids were purchased from the Korean Cell Line Bank (Seoul, Republic of Korea) and maintained at 37 °C in 5% CO_2_/95% air in Advanced DMEM:F12 complete medium supplemented with HEPES (10 mM), L-glutamine (2 mM), Noggin (100 ng/mL), human fibroblast growth factor 10 (100 ng/mL), nicotinamide (10 mM), N-acetyl cysteine (1.25 mM), 1× B-27, human epidermal growth factor (50 ng/mL), A83-01 (500 nM), gastrin (10 nM), Wnt-3A (120 ng/ml), and R-spondin1 (175 ng/mL). Organoids were passaged approximately every 7 days by dissociation using TrypLE (Gibco, USA) for 10 min at 37 °C. Cells were resuspended in growth factor-reduced Matrigel (Corning, USA) and seeded as 50-μL drops into 24-well plates. After a 15-min incubation period at 37 °C, 1 mL of organoid growth medium was added.

### ATP-Glo cell viability assay

Pancreatic cancer organoids were harvested with a scraper and dissociated into single cells using Trypsin-EDTA. The procedure was performed using an ATP-Glo cell viability assay kit (Promega). All measurements were performed in accordance with the manufacturer's protocol using a Luminometer (Perkin Elmer, USA).

### Patient data and bioinformatic analysis

RNA expression and copy number variation (CNV) data for pancreatic adenocarcinoma (TCGA-PAAD) were obtained from UCSC XenaBrowser (https://xena.ucsc.edu). Data from a total of 91 patients were included. Patients were stratified into high (top 25%) and low (bottom 25%) groups based on CHRNA7 mRNA expression and CNV status. Overall survival between groups was analyzed using Kaplan-Meier curves and the log-rank test.

### Animal experiments

The animal experiments in this study were approved by the Institutional Animal Care and Use Committee of Asan Institute for Life Science (IACUC Number: 2025-40-236). Tumors were generated by subcutaneously injecting AsPC-1/pshCont or AsPC-1/pshnAChR-α7 cells (2 × 10^6^ cells/mouse) into the right flank of mice. Injected mice were assigned to groups (3-4 mice per group) and administered acetylcholine (5 mg/kg) by tail vein injection 2 times a week for 45 d. Tumor size was measured every 3 to 7 d using a digital caliper, and tumor volume (TV) was calculated using the following formula: TV = length × (width)^2^ × 0.5. Per IACUC guidelines, mice were euthanized after 45 d and tumors were harvested.

### Immunohistochemistry

Tissues were fixed in buffered formalin and subsequently embedded in paraffin. Paraffin blocks were sectioned at a thickness of 4 μm, followed by dewaxing and rehydration through a graded ethanol series. Antigen retrieval was carried out by heating the slides in 10 mM citrate buffer (pH 6.0) using a microwave oven for 14 min. Endogenous peroxidase activity was quenched by incubating with 3% hydrogen peroxide in PBS for 10 min. Sections were then incubated overnight at 4°C with anti-nAChR-α7 (1:100, #AB216485; Abcam), anti-Ki-67 (1:100, #12202S, Cell Signaling Technology), anti-cleaved caspase 3 (1:100, #9664S, Cell Signaling Technology), and anti-HIF-1α (1:1000, #sc-13515, Santa Cruz Biotechnology). Immunohistochemistry images were obtained at 20 × magnification using a Lionheart FX Automated Microscope (BioTek Instruments, USA), and representative images were recorded.

### Statistical analysis

All error bars represent means ± SEM. Differences between groups were analyzed by unpaired Student's *t* test. Statistical analyses were performed using GraphPad Prism software (GraphPad, USA). A P-value < 0.05 was considered statistically significant; individual P-values (*P < 0.05, **P < 0.01, ***P < 0.001, ****P < 0.0001; NS, not significant) are indicated in figure legends.

## Results

### Acetylcholine enhances HIF-1α expression in pancreatic cancer cells under hypoxic conditions

**Figure 1:**
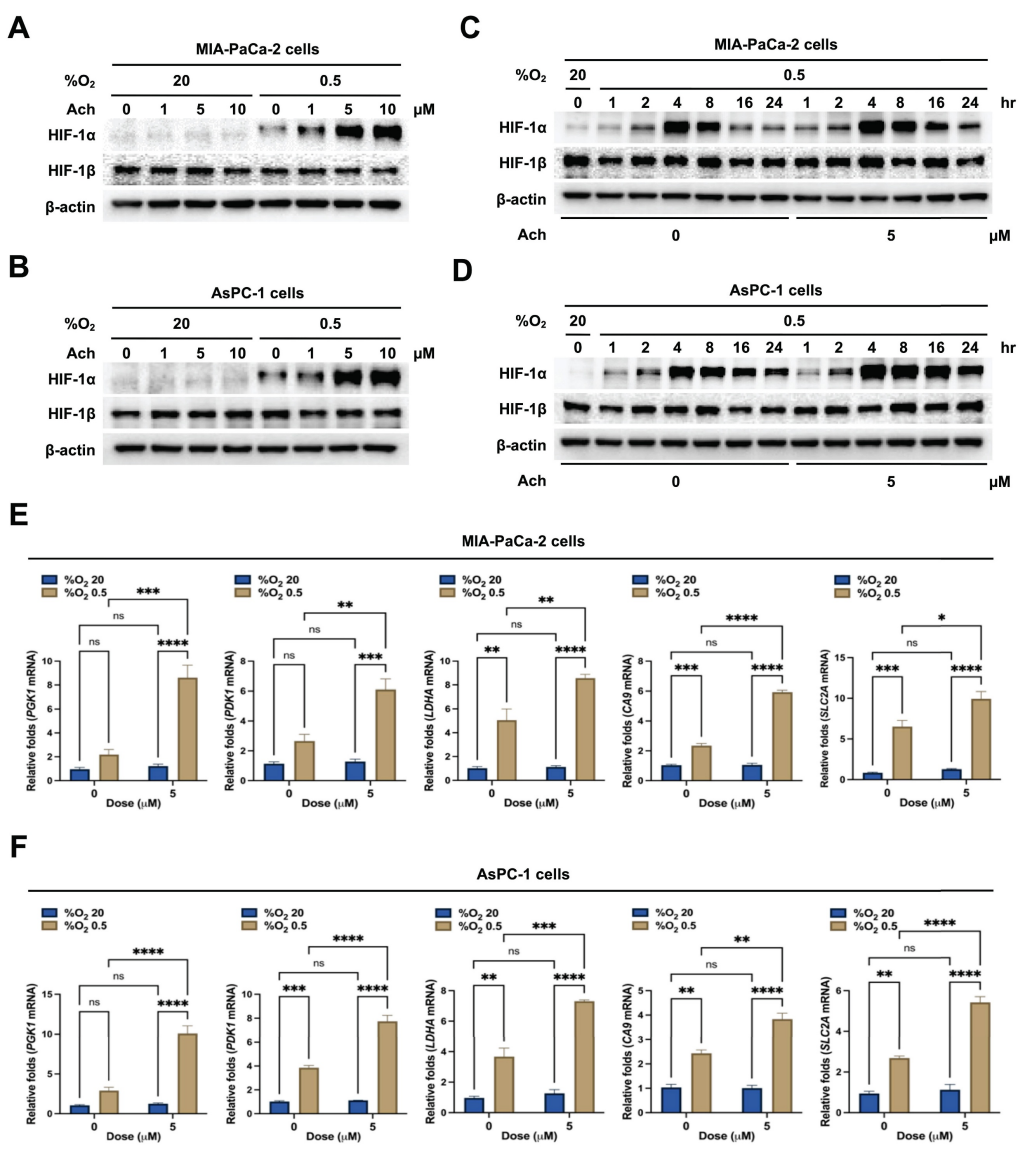
Acetylcholine enhances HIF-1α expression in pancreatic cancer cells exposed to hypoxia. **(A, B)** MIA-PaCa-2 **(A)** and AsPC-1 **(B)** cells were treated with a range of concentrations of acetylcholine (0-10 μM) for 1 h and exposed to 20% or 0.5% O_2_. After a 24-h incubation period, cells were harvested, and whole-cell lysates were analyzed for the indicated proteins by immunoblotting. **(C, D)** MIA-PaCa-2 **(C)** and AsPC-1 **(D)** cells were treated with 5 μM acetylcholine or left untreated for 1 h and exposed to 20% or 0.5% O_2_. After a 24-h incubation period, cells were harvested, and whole-cell lysates were analyzed for the indicated proteins by immunoblotting. **(E, F)** MIA-PaCa-2 **(E)** and AsPC-1 **(F)** cells were treated with 5 μM acetylcholine or left untreated for 1 h, exposed to 20% or 0.5% O_2_ for 24 h, and harvested. qPCR was employed to amplify *PGK1*, *PDK1*, *LDHA*, *CA9*, and *SLC2A* using *18S rRNA* as an internal control. Data are presented as means ± SEMs (**P < 0.01, ***P < 0.001, ****P < 0.0001; ANOVA). Ns indicates no significance.

To establish the effect of acetylcholine on HIF-1α expression in pancreatic cancer cells under hypoxic conditions, we treated MIA-PaCa-2 and AsPC-1 cells with acetylcholine (0-10 μM) for 1 h, followed by exposure to 20% or 0.5% O_2_ for 24 h. Expression levels of HIF-1α and HIF-1β were assessed by immunoblot analysis. Acetylcholine induced a concentration-dependent increase in HIF-1α expression in both MIA-PaCa-2 and AsPC-1 cells in hypoxia, producing a maximal effect at concentrations > 5 μM, but did not alter HIF-1β expression (**Figure 1A, B**). To confirm these findings, we treated MIA-PaCa-2 and AsPC-1 cells with 5 μM acetylcholine or left them untreated for 1 h, then exposed them to 20% or 0.5% O_2_ for 24 h, and assessed HIF-1α and HIF-1β expression by immunoblot analysis. Notably, HIF-1α levels were markedly increased in both cell types in a 0.5% O_2_ environment, displaying a peak in expression at 4 h followed by a gradual decline (**Figure 1C, D**). Treatment with 5 μM acetylcholine delayed the decrease in HIF-1α expression by 24 h in both cell types under hypoxic conditions (**Figure 1C, D**). However, HIF-1β levels remained unaffected, regardless of oxygen concentration or acetylcholine treatment (**Figure 1C, D**). Next, we evaluated the effects of acetylcholine on the transcriptional activity of HIF-1α, which regulates numerous hypoxia-adaptive genes containing hypoxia response elements (HREs) in their promoter regions 25. MIA-PaCa-2 and AsPC-1 cells were treated with 5 μM acetylcholine or left untreated for 24 h, and expression patterns of HIF-1α target genes were analyzed by qPCR. Notably, treatment with acetylcholine under hypoxic conditions enhanced expression of the target genes, *SLC2A*, *LDHA*, *PDK1*, *PGK1* and *CA9*, in pancreatic cancer cells (**Figure 1E, F**). To validate these findings, we transfected MIA-PaCa-2 and AsPC-1 cells with a reporter plasmid (p3×HRE-*luc*) and pRL-*luc* (transfection control), treated them with 5 μM acetylcholine or left them untreated, and exposed them to 20% or 0.5% O_2_ for 24 h. Under hypoxic conditions, acetylcholine enhanced luciferase activity in pancreatic cancer cells transfected with the reporter plasmid compared with cells in the normoxic control group (**Supplementary Figure S1**). Collectively, these data indicate that acetylcholine treatment enhances HIF-1α expression, thereby increasing HIF-1α-mediated transcription and expression of downstream target genes.

### Acetylcholine enhances HIF-1α expression in pancreatic cancer cells in hypoxia through nAChR-α7

**Figure 2:**
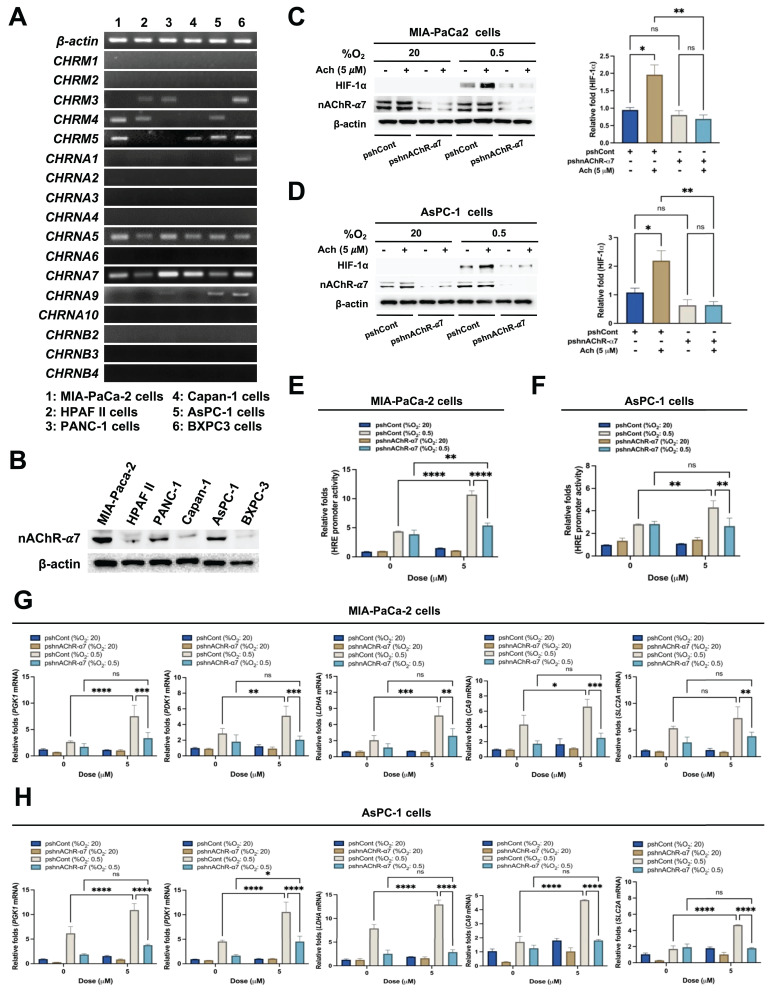
Acetylcholine enhances HIF-1α expression via nAChR-α7 in pancreatic cancer cells exposed to hypoxia. **(A)** Following harvest of pancreatic cancer cells (MIA-PaCa-2, HPAF-II, PANC-1, Capan-1, AsPC-1 and BxPC3), the indicated genes and *18S rRNA* were amplified by RT-PCR. Band intensities of RT-PCR products from cells were analyzed using the ChemiDoc imaging system. **(B)** Pancreatic cancer cells were harvested and whole-cell lysates analyzed by immunoblotting for nAChR-α7. **(C, D)** pshCont- or pshnAChR-α7-transfected MIA-PaCa-2 **(C)** and AsPC-1 **(D)** cells were treated with 5 μM acetylcholine or left untreated for 1 h, exposed to 20% or 0.5% O_2_ for 24 h, and harvested. The indicated proteins in whole-cell lysates were analyzed by immunoblotting. HIF-1α protein signals were detectable and quantified only under 0.5% O_2_ conditions. Left panels: Representative immunoblot images for HIF-1α protein levels. Right panels: Band intensities of HIF-1α, obtained utilizing data from both Figure 2C, D and Supplementary Figure S2 (three independent biological replicates). Results, quantified using Image J software and normalized to β-actin, are presented as means ± SEM (*P < 0.05, **P < 0.01; ANOVA). Ns indicates no significance. **(E, F)** pshCont- or pshnAChR-α7-transfected MIA-PaCa-2 **(E)** and AsPC-1 **(F)** cells were co-transfected with p3×HRE-*luc* and pRL-*luc*, incubated with 5 μM acetylcholine or left untreated for 1 h, and then exposed to 20% or 0.5% O_2_ for 24 h. Luciferase activity in whole-cell lysates was normalized to that of *Renilla* luciferase. Data are presented as means ± SEM (**P < 0.01, ****P < 0.0001; ANOVA). Ns indicates no significance. **(G, H)** pshCont- or pshnAChR-α7-transfected MIA-PaCa-2 **(G)** and AsPC-1 **(H)** cells were incubated with 5 μM acetylcholine or left untreated for 1 h, then exposed to 20% or 0.5% O_2_ for 24 h and harvested, after which HIF-1α target genes (*PGK1*, *PDK1*, *LDHA*, *CA9*, and *SLC2A*) were examined by qPCR using *18S rRNA* as an internal control. Data are presented as means ± SEMs (*P < 0.05, **P < 0.01, ***P < 0.001, ****P < 0.0001; ANOVA). Ns indicates no significance.

Next, we focused on the receptors associated with acetylcholine-induced enhancement of HIF-1α expression in hypoxic pancreatic cancer cells. mRNA expression levels of the five mAChR subtypes, M1-M5 (encoded by *CHRM1*-*CHRM5*), were examined in the pancreatic cancer lines MIA-PaCa-2, HPAF-II, PANC-1, Capan-1, AsPC-1, and BxPC-3 using RT-PCR. mRNA levels of nAChR α-subunits 1-7, 9 and 10 (encoded by *CHRNAs* 1-7, 9 and 10), and nAChR β-subunits 2-4 (encoded by *CHRNBs* 2-4) were also analyzed in these cells. All pancreatic cancer cell lines expressed *CHRNA5* and *CHRNA7*, as shown in **Figure 2A**. As reported previously, nAChR-α5 is a heteromeric form of nAChR 47. Given the expression of both* CHRNA5* and *CHRNA7* mRNA, we further examined whether the effects of acetylcholine on HIF-1α expression could be attributable to nAChR-α7. Consistent with RT-PCR findings, nAChR-α7 protein expression was evident in all pancreatic cancer cells (**Figure 2B**). MIA-PaCa-2 and AsPC-1 cells were transfected with plasmids expressing short hairpin RNAs (shRNAs) targeting AChR-α7 (pshnAChR-α7) or with non-targeting pshRNA (pshCont), treated with 5 μM acetylcholine or left untreated for 1 h, and subsequently exposed to 20% or 0.5% O_2_. After a 24 h incubation, cells were harvested and HIF-1α expression was assessed by immunoblot analysis. Notably, shRNA-mediated knockdown of nAChR-α7 inhibited acetylcholine-induced enhancement of HIF-1α expression in pancreatic cancer cells under hypoxic conditions (**Figure 2C, D** and** Supplementary Figure S2**). To validate these findings, we transfected MIA-PaCa-2/pshCont, MIA-PaCa-2/pshnAChR-α7, AsPC-1/pshCont and AsPC-1/pshnAChR-α7 cells with p3×HRE-*luc* and pRL-*luc*, followed by treatment with or without 5 μM acetylcholine and exposure to 20% or 0.5% O_2_ for 24 h. Acetylcholine enhanced luciferase activity in pancreatic cancer cells under hypoxic conditions, an effect that was inhibited by knockdown of nAChR-α7 (**Figure 2E, F**). To further substantiate these results, we treated MIA-PaCa-2/pshCont, MIA-PaCa-2/pshnAChR-α7, AsPC-1/pshCont and AsPC-1/pshnAChR-α7 cells with 5 μM acetylcholine or left them untreated for 1 h, and subsequently exposed them to 20% or 0.5% O_2_. After incubating for 24 h, cells were harvested and expression of HIF-1α target genes was analyzed using qPCR. Interestingly, suppression of nAChR-α7 inhibited acetylcholine-induced enhancement of the HIF-1α target genes, *SLC2A*, *LDHA*, *PDK1*, *PGK1* and *CA9*, in hypoxia-exposed pancreatic cancer cells (**Figure 2G, H**). We further explored whether acetylcholine could enhance nAChR-α7-mediated HIF-1α expression in pancreatic cancer cells in hypoxia by treating cells with the mAChR-specific inhibitor, scopolamine, or the nAChR-α7-specific inhibitor, α-bungarotoxin. As shown in **Supplementary Figure S3**, scopolamine had no effect on acetylcholine-induced enhancement of HIF-1α in pancreatic cancer cells under hypoxic conditions. In contrast, α-bungarotoxin inhibited acetylcholine-induced enhancement of HIF-1α in a concentration-dependent manner (**Supplementary Figure S4**). Collectively, these results suggest that acetylcholine enhances HIF-1α expression via nAChR-α7 in pancreatic cancer cells under hypoxic conditions.

### Acetylcholine increases HIF-1α protein stability in hypoxia-exposed pancreatic cancer cells

**Figure 3:**
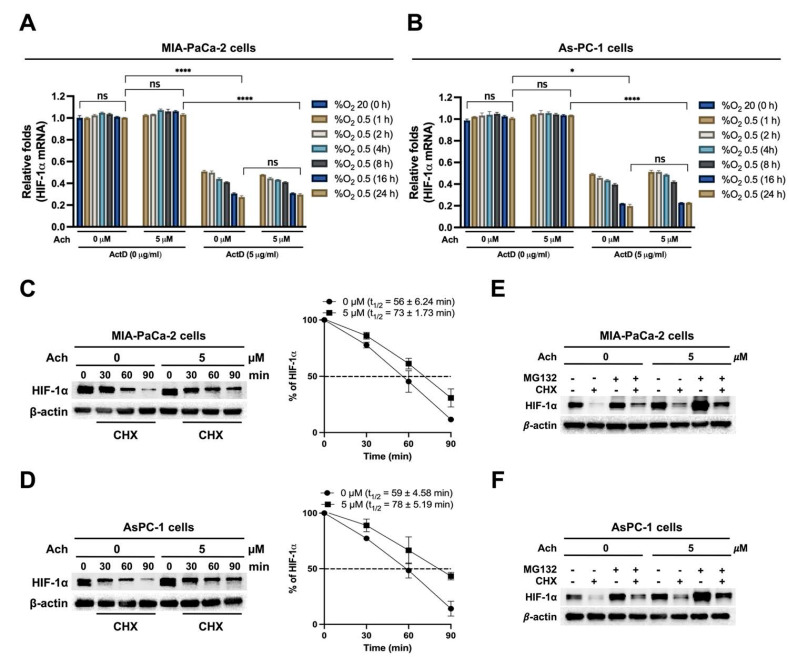
Acetylcholine increases HIF-1α protein stability in pancreatic cancer cells exposed to hypoxia. **(A, B)** MIA-PaCa-2 **(A)** and AsPC-1 **(B)** cells were treated with or without 5 μM actinomycin D (ActD) for 1 h, followed by treatment with or without 5 μM acetylcholine (Ach) for 1 h. Cells were then cultured under hypoxic conditions (0.5% O_2_) for the indicated time periods (1, 2, 4, 8, 16, and 24 h). Cells maintained under normoxic conditions (20% O_2_) without any treatment served as the control group. *HIF-1α* mRNA levels were determined by qPCR using *18S rRNA* as an internal control. Data are presented as means ± SEMs (*P < 0.05, ****P < 0.0001; ANOVA). Ns indicates no significance. **(C, D)** MIA-PaCa-2 **(C)** and AsPC-1 **(D)** cells were treated with 5 μM acetylcholine or left untreated for 1 h and exposed to 20% or 0.5% O_2_. After a 24-h incubation period, cells were treated with 10 μg/mL CHX or left untreated and harvested at the indicated times. HIF-1α protein levels were examined by immunoblot analysis using β-actin as an internal control. Left panel: Representative images of immunoblot analysis. Right panel: HIF-1α protein levels, measured as band intensities, quantified using Image J and normalized to that of β-actin (band intensity at t_0_ is defined as 100%). **(E, F)** MIA-PaCa-2 **(E)** and AsPC-1 **(F)** cells were treated with 5 μM acetylcholine or left untreated for 1 h and exposed to 20% or 0.5% O_2_. After a 24-h incubation period, cells were treated with MG132 or CHX or left untreated for 90 min. Cells were harvested, and the indicated proteins in whole-cell lysates were analyzed by immunoblotting.

To elucidate the mechanisms underlying acetylcholine-induced upregulation of HIF-1α expression via nAChR-α7 in pancreatic cancer cells in hypoxia, we examined *HIF-1α* mRNA expression and stability. MIA-PaCa-2 and AsPC-1 cells were pretreated with or without 5 μg/mL actinomycin D (ActD) for 1 h and treated with or without 5 μM acetylcholine for 1 h. Cells were then cultured under hypoxic conditions (0.5% O_2_) for different periods (1, 2, 4, 8, 16, and 24 h). Untreated cells maintained under normoxia (20% O_2_) were used as controls. Following harvest of cells at the indicated times, *HIF-1α* mRNA expression was examined by qPCR. This analysis revealed that *HIF-1α* mRNA expression was unaffected by acetylcholine treatment in pancreatic cancer cells under hypoxic conditions (0.5% O_2_) (**Figure 3A, B**). Moreover, ActD pretreatment led to comparable decreases in both acetylcholine-treated and untreated cells, indicating that acetylcholine does not influence HIF-1α mRNA stability under hypoxic conditions (**Figure 3A, B**). We subsequently investigated whether acetylcholine affects the accumulation and stability of HIF-1α protein in pancreatic cancer cells in hypoxia. As shown in **Figure 1C, D**, HIF-1α expression peaked at 4 h in pancreatic cancer cells exposed to hypoxia, with or without acetylcholine treatment; thus, this time point was selected for subsequent analyses of protein stability. MIA-PaCa-2 and AsPC-1 cells were treated with 5 μM acetylcholine or left untreated for 1 h, and exposed to 0.5% O_2_ for 4 h. Cells were further treated with 10 μg/mL cycloheximide (CHX), to block new protein synthesis, or left untreated, and incubated for 1.5 h under hypoxic conditions. After harvesting cells at the indicated times, we examined the decrease in HIF-1α protein over time by immunoblot analysis. This analysis showed that acetylcholine further delayed CHX-induced HIF-1α degradation in a time-dependent manner compared with CHX alone. Notably, degradation of HIF-1α was further delayed in a time-dependent manner upon simultaneous treatment with CHX and acetylcholine compared to treatment with CHX alone (**Figure 3C, D**). Because endogenous HIF-1α protein levels were noticeably different at longer time points (16 and 24 h) in hypoxia-exposed pancreatic cancer cells (**Figure 1C, D**), we used exogenously expressed HA-tagged HIF-1α (HA-HIF-1α) to more accurately assess HIF-1α stability at these time points. First, MIA-PaCa-2 cells were transfected with siRNA targeting HIF-1α (siHIF-1α) or non-targeting control siRNA (siCont), with or without pHA-HIF-1α, for 24 h. After exposure to hypoxic conditions (0.5% O_2_) for 24 h, HIF-1α expression in cells was analyzed by immunoblot analysis. As expected, siHIF-1α effectively knocked down HIF-1α expression in cells under hypoxic conditions (0.5% O_2_); we further found that overexpression of HA-HIF-1α rescued siHIF-1α-induced inhibition of HIF-1α expression (**Supplementary Figure S5A**). We then used HA-HIF-1α to monitor HIF-1α stability over time. To this end, MIA-PaCa-2 and AsPC-1 cells were transfected with siHA-HIF-1α and pHA-HIF-1α for 24 h, treated with or without 5 μM acetylcholine, and exposed to 0.5% O_2_ for the indicated times (1, 2, 4, 8, 16, and 24 h). Following harvest of cells at the indicated time points, HIF-1α expression was assessed using immunoblot analysis. This analysis revealed that HA-HIF-1α expression reached its maximum at 16 h in cells under hypoxic conditions (0.5% O_2_) (**Supplementary Figure S5B**). Using this time point, CHX chase assays demonstrated that acetylcholine markedly slowed degradation of HA-HIF-1α compared with CHX treatment alone (**Supplementary Figure S5C**). These results confirm that acetylcholine promotes the sustained stabilization of HIF-1α protein in pancreatic cancer cells under hypoxic conditions. Previous findings suggest that the proteasome system regulates HIF-1α degradation 25. To establish whether acetylcholine suppresses proteasome-mediated degradation of HIF-1α in hypoxia, we treated MIA-PaCa-2 and AsPC-1 cells with 5 μM acetylcholine or left them untreated for 1 h, then exposed them to 0.5% O_2_ for 4 h and treated them with or without the proteasome inhibitor, MG132, or CHX. After 90 min, cells were harvested and HIF-1α levels were assessed by immunoblot analysis. MG132 treatment inhibited the degradation of HIF-1α in pancreatic cancer cells treated with CHX in a hypoxic environment. Notably, acetylcholine protected against proteasome-mediated degradation of HIF-1α induced by CHX (**Figure 3E, F**), clearly indicating a stabilization effect on HIF-1α protein in hypoxic pancreatic cancer cells.

### Acetylcholine promotes YAP nuclear localization and HIF-1α signaling in hypoxia-exposed pancreatic cancer cells

**Figure 4:**
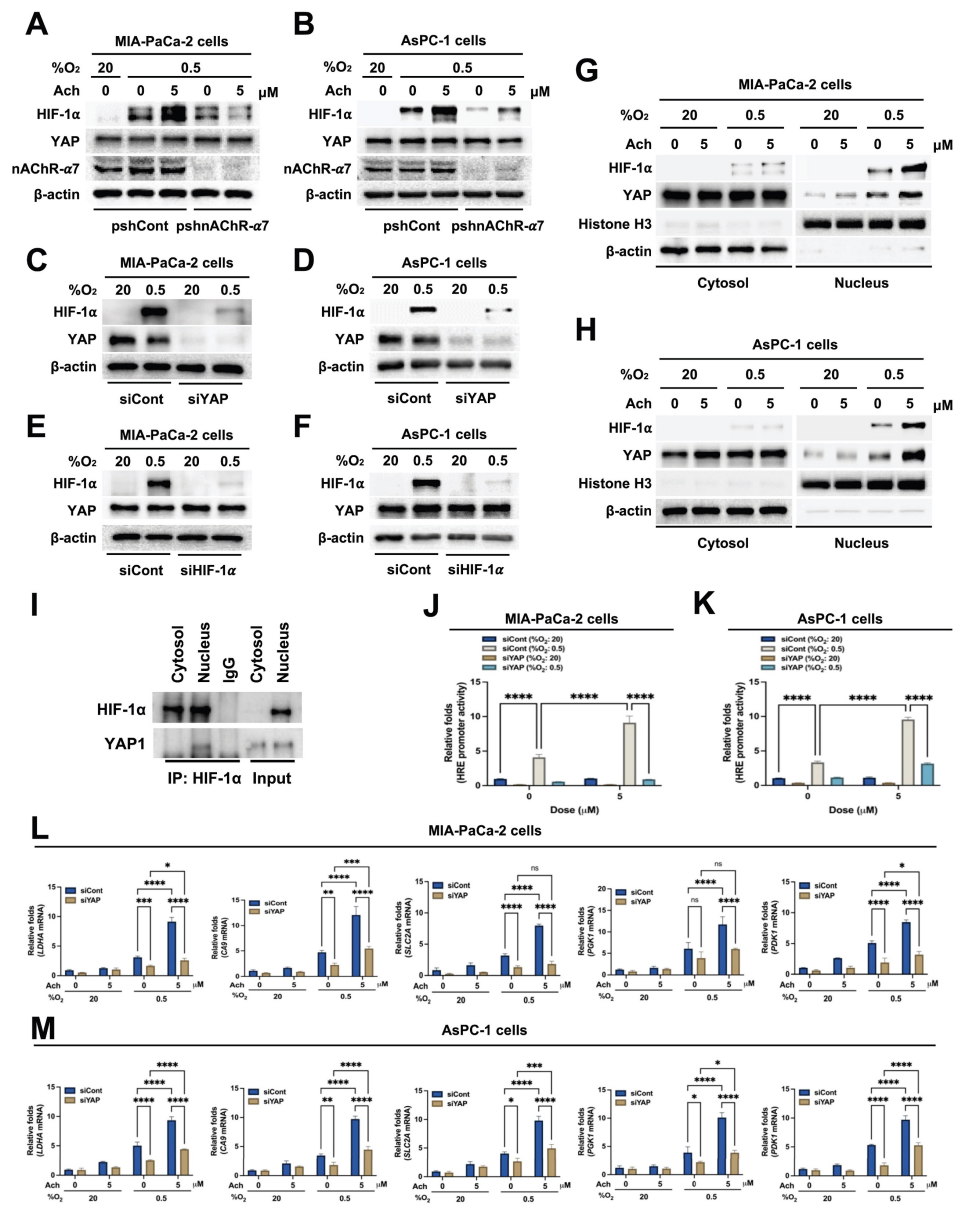
Acetylcholine promotes localization of YAP and enhances the HIF-1α signaling pathway in pancreatic cancer cells exposed to hypoxia. **(A, B)** pshCont- or pshnAChR-α7-transfected MIA-PaCa-2 **(A)** and AsPC-1 **(B)** cells were treated with 5 μM acetylcholine or left untreated for 1 h and exposed to 20% or 0.5% O_2_. After a 24-h incubation period, cells were harvested and the indicated proteins in whole-cell lysates were analyzed by immunoblotting. **(C, D)** MIA-PaCa-2 **(C)** and AsPC-1 **(D)** cells were transfected with siCont or siYAP, incubated for 48 h, and exposed to 20% or 0.5% O_2_. After a 24-h incubation period, cells were harvested and the indicated proteins in whole-cell lysates were analyzed by immunoblotting. **(E, F)** MIA-PaCa-2 **(E)** and AsPC-1 **(F)** cells were transfected with siCont or siHIF-1α, incubated for 48 h, and exposed to 20% or 0.5% O_2_. After 24 h of incubation, cells were harvested, and the indicated proteins in whole-cell lysates were analyzed by immunoblotting. **(G, H)** MIA-PaCa-2 **(G)** and AsPC-1 **(H)** cells were treated with 5 μM acetylcholine or left untreated for 1 h, followed by exposure to 20% or 0.5% O_2_. After a 24-h incubation period, cells were harvested for preparation of nuclear and cytoplasmic protein extracts, and the indicated proteins were analyzed by immunoblotting. **(I)** Whole-cell extracts of MIA-PaCa-2 cells were immunoprecipitated with anti-HIF-1α or anti-IgG (negative control), followed by immunoblot analysis with anti-HIF-1α and anti-YAP antibodies. **(J, K)** siCont- or siYAP-transfected MIA-PaCa-2 **(J)** and AsPC-1 **(K)** cells were co-transfected with p3×HRE-*luc* and pRL-*luc*, followed by treatment with or without 5 μM acetylcholine for 1 h, and exposure to 20% or 0.5% O_2_ for 24 h. Luciferase activity measured in whole-cell lysates was normalized to that of *Renilla* luciferase. Data are presented as means ± SEM (****P < 0.0001; ANOVA). **(L, M)** siCont- or siYAP-transfected MIA-PaCa-2 **(L)** and AsPC-1 **(M)** cells were incubated with 5 μM acetylcholine or left untreated for 1 h and exposed to 20% or 0.5% O_2_ for 24 h. The HIF-1α target genes, *PGK1*, *PDK1*, *LDHA*, *CA9* and *SLC2A,* in harvested cells were examined by qPCR using *18S rRNA* as an internal control. Data are presented as means ± SEM (*P < 0.05, **P < 0.01, ***P < 0.001, ****P < 0.0001; ANOVA). Ns indicates no significance.

Earlier research has shown that hypoxia induces the translocation of YAP from the cytoplasm to the nucleus in hepatocellular carcinoma cells 21. Nuclear YAP binds to HIF-1α, thereby enhancing its protein stability 21. We initially investigated whether acetylcholine acts through upregulation of YAP to enhance nAChR-α7-mediated HIF-1α expression in pancreatic cancer cells under hypoxic conditions. To this end, MIA-PaCa-2/pshCont, MIA-PaCa-2/pshnAChR-α7, AsPC-1/pshCont and AsPC-1/pshnAChR-α7 cells were treated with 5 μM acetylcholine or left untreated for 1 h, followed by exposure to 0.5% O_2_ for 24 h. Subsequently, cells were harvested, and nAChR-α7, HIF-1α and YAP expression levels were determined by immunoblot analysis. As shown in **Figure 4A, B**, knockdown of nAChR-α7 suppressed acetylcholine-induced enhancement of HIF-1α expression in hypoxic pancreatic cancer cells, without affecting YAP expression. Next, we focused on whether YAP upregulates HIF-1α expression in pancreatic cancer cells in hypoxia. For this experiment, MIA-PaCa-2 and AsPC-1 cells were transfected with siCont or siYAP and exposed to 0.5% O_2_ for 24 h. Immunoblot analyses revealed that siYAP significantly inhibited the expression of YAP and HIF-1α (**Figure 4C, D**). We further examined the potential regulatory effects of HIF-1α on YAP expression in pancreatic cancer cells under hypoxic conditions by knocking down HIF-1α with siHIF-1α. To this end, MIA-PaCa-2 and AsPC-1 cells were transfected with siCont or siHIF-1α and exposed to 0.5% O_2_ for 24 h. Notably, siHIF-1α significantly inhibited HIF-1α expression in hypoxic pancreatic cancer cells, but did not affect YAP expression (**Figure 4E, F**). In view of these results, we hypothesized that acetylcholine promotes translocation of YAP from the cytoplasm to the nucleus in hypoxia-exposed pancreatic cancer cells, which, in turn, elevates HIF-1α expression. As illustrated in **Figure 4G, H**, acetylcholine enhanced hypoxia-induced translocation of YAP and HIF-1α in pancreatic cancer cells. We next examined how acetylcholine-induced nuclear YAP binds to HIF-1α and enhances its expression under hypoxic conditions. Using co-immunoprecipitation, we found that HIF-1α binds to YAP in the nucleus (**Figure 4I**). To substantiate these results, we transfected MIA-PaCa-2 and AsPC-1 cells first with siCont or siYAP and then with p3×HRE-*luc* and pRL-*luc*, treated them with 5 μM acetylcholine or left them untreated, and subsequently exposed them to 20% or 0.5% O_2_ for 24 h. siYAP inhibited acetylcholine-induced enhancement of luciferase activity in hypoxic pancreatic cancer cells to a greater extent than in normoxic control cells (**Figure 4J, K**).

For further confirmation, MIA-PaCa-2 and AsPC-1 cells were transfected with siCont or siYAP, treated with 5 μM acetylcholine or left untreated for 1 h, and subsequently exposed to 20% or 0.5% O_2_. After 24 h of incubation, cells were harvested and expression of HIF-1α target genes was analyzed using qPCR. Suppression of YAP inhibited acetylcholine-induced enhancement of the HIF-1α target genes, *SLC2A*, *LDHA*, *PDK1*, *PGK1* and *CA9* (**Figure 4L, M**). Collectively, our results indicate that acetylcholine stimulates translocation of YAP from the cytoplasm to the nucleus of pancreatic cancer cells in hypoxia, thereby promoting HIF-1α signaling.

### Acetylcholine enhances HIF-1α signaling in hypoxic pancreatic cancer cells through regulation of PDPK1-mediated translocation of YAP

**Figure 5:**
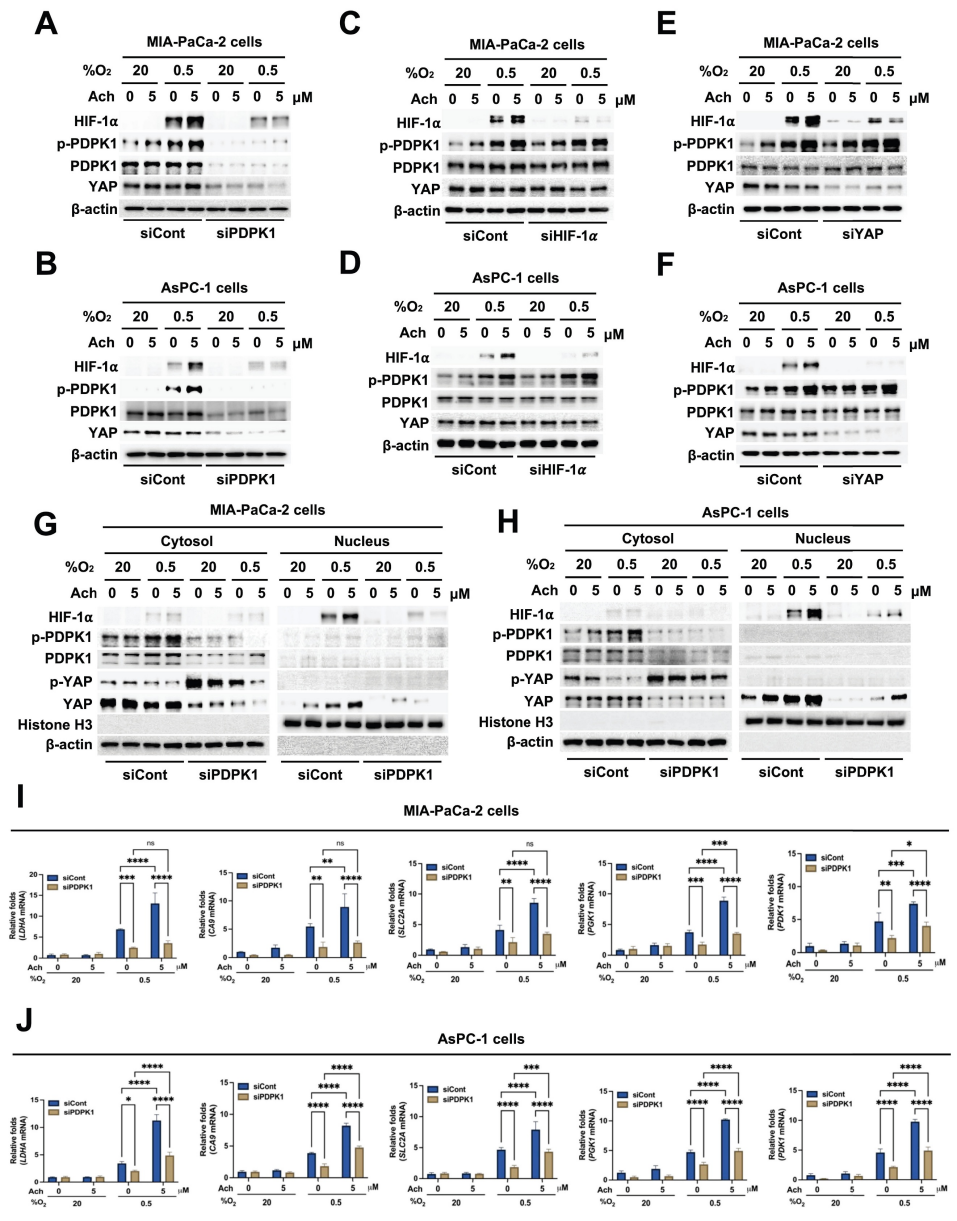
Acetylcholine enhances HIF-1α signaling through regulation of PDPK1-mediated translocation of YAP in pancreatic cancer cells exposed to hypoxia. **(A, B)** siCont- or siPDPK1-transfected MIA-PaCa-2 **(A)** and AsPC-1 **(B)** cells were treated with 5 μM acetylcholine or left untreated for 1 h and exposed to 20% or 0.5% O_2_. After a 24-h incubation period, cells were harvested and the indicated proteins were analyzed in whole-cell lysates by immunoblotting. **(C, D)** MIA-PaCa-2 **(C)** and AsPC-1 **(D)** cells were transfected with siCont or siHIF-1α, incubated for 48 h, treated with 5 μM acetylcholine or left untreated for 1 h, and exposed to 20% or 0.5% O_2_. After a 24-h incubation, cells were harvested and the indicated proteins in whole-cell lysates were analyzed by immunoblotting. **(E, F)** MIA-PaCa-2 **(E)** and AsPC-1 **(F)** cells were transfected with siCont or siYAP, incubated for 48 h, treated with 5 μM acetylcholine or left untreated for 1 h, and exposed to 20% or 0.5% O_2_. After a 24-h incubation period, cells were harvested and the indicated proteins in whole-cell lysates were analyzed by immunoblotting. **(G, H)** MIA-PaCa-2 **(G)** and AsPC-1 **(H)** cells were treated with 5 μM acetylcholine or left untreated for 1 h and exposed to 20% or 0.5% O_2_. After a 24-h incubation period, cells were harvested and used to prepare nuclear and cytoplasmic protein extracts, followed by immunoblot analysis of the indicated proteins. **(I, J)** siCont- or siPDPK1-transfected MIA-PaCa-2 **(I)** and AsPC-1 **(J)** cells were incubated with 5 μM acetylcholine or left untreated for 1 h, followed by exposure to 20% or 0.5% O_2_ for 24 h. Levels of HIF-1α target genes (*PGK1*, *PDK1*, *LDHA*, *CA9*, and *SLC2A*) were examined in harvested cells by qPCR using *18S rRNA* as the internal control. Data are presented as means ± SEM (*P < 0.05, **P < 0.01, ***P < 0.001, ****P < 0.0001; ANOVA). Ns indicates no significance.

Activation of PDPK1 has previously been shown to reduce phosphorylation of YAP (Ser 397), leading to its translocation from the cytosol to the nucleus 36. Therefore, we investigated whether acetylcholine activates PDPK1 in pancreatic cancer cells in hypoxia, subsequently promoting nuclear translocation of YAP and thereby enhancing HIF-1α expression. MIA-PaCa-2 and AsPC-1 cells were transfected with siCont or siPDPK1, treated with 5 μM acetylcholine or left untreated for 1 h, and exposed to 0.5% O_2_ for 24 h. An immunoblot analysis of HIF-1α, YAP and PDPK1 expression levels in harvested cells showed that siPDPK1 suppressed the acetylcholine-induced enhancement of HIF-1α and YAP expression in hypoxic pancreatic cancer cells (**Figure 5A, B**). To further ascertain the involvement of acetylcholine in activation of PDPK1 in these cells under hypoxic conditions, we examined downstream targets of PDPK1. Notably, acetylcholine activated the PDPK1 target proteins, AKT and S6K, in pancreatic cancer cells under hypoxic conditions, actions that were effectively inhibited by siPDPK1 (**Supplementary Figure S6**). Next, we explored whether acetylcholine-induced enhancement of HIF-1α and YAP expression impacts the activation and expression of PDPK1 in pancreatic cancer cells under hypoxic conditions. Specifically, MIA-PaCa-2 and AsPC-1 cells were transfected with siCont or siHIF-1α, treated with 5 μM acetylcholine or left untreated for 1 h, and exposed to 0.5% O_2_ for 24 h. Our results showed that siHIF-1α significantly inhibited HIF-1α expression in pancreatic cancer cells in the absence or presence of acetylcholine, but had no effects on PDPK1 expression or activation in pancreatic cancer cells in hypoxia (**Figure 5C, D**). In addition, siHIF-1α did not inhibit YAP expression in pancreatic cancer cells in the absence or presence of acetylcholine (**Figure 5C, D**). Next, MIA-PaCa-2 and AsPC-1 cells were transfected with siCont or siYAP, treated with 5 μM acetylcholine or left untreated for 1 h, and exposed to 0.5% O_2_ for 24 h. Notably, siYAP inhibited YAP and HIF-1α expression, both in the absence and presence of acetylcholine, but exerted no effects on PDPK1 expression or activity in hypoxic pancreatic cancer cells (**Figure 5E, F**). In view of these results, we tested the hypothesis that acetylcholine-induced activation of PDPK1 leads to a reduction in serine 397 phosphorylation of YAP, thereby promoting its translocation to the nucleus and leading to elevated HIF-1α expression. As shown in **Figure 5G, H**, acetylcholine-induced PDPK1 activation enhanced HIF-1α expression, reduced phosphorylation at serine 397, and promoted accumulation of cytosolic YAP and nuclear HIF-1α. To confirm these findings, we transfected MIA-PaCa-2 and AsPC-1 cells first with siCont or siPDPK1 and then with p3×HRE-*luc* and pRL-*luc*, treated them with 5 μM acetylcholine or left them untreated, and subsequently exposed them to 20% or 0.5% O_2_ for 24 h. siPDPK1 inhibited acetylcholine-induced enhancement of luciferase activity relative to the control group (**Supplementary Figure S7**). For further validation, MIA-PaCa-2 and AsPC-1 cells transfected with siCont or siPDPK1 were treated with 5 μM acetylcholine or left untreated for 1 h and exposed to 20% or 0.5% O_2_. After 24 h of incubation, cells were harvested and expression of HIF-1α target genes was analyzed using qPCR. Suppression of PDPK1 inhibited acetylcholine-induced enhancement of the HIF-1α target genes, *SLC2A*, *LDHA*, *PDK1*, *PGK1* and *CA9*, in hypoxia-exposed pancreatic cancer cells (**Figure 5I, J**). In pancreatic cancer, nicotine activates nAChR-α7, leading to Src-mediated PI3K signaling 45. PI3K, in turn, activates PDPK1, resulting in reduced intracellular phosphorylation of YAP at serine 397 36. Accordingly, we investigated whether acetylcholine acts through nAChR-α7 to enhance activation of PI3K and thereby regulate the PDPK1/YAP/HIF-1α signaling axis in pancreatic cancer cells exposed to hypoxia. As illustrated in **Supplementary Figure S8**, pshnAChR-α7 inhibited the acetylcholine-induced increase in PI3K activation, leading to inhibition of the PDPK1/YAP/HIF-1α signaling pathway in these hypoxic cells. Our data indicate that acetylcholine facilitates translocation of YAP from the cytosol to the nucleus, thereby enhancing HIF-1α expression in hypoxic pancreatic cancer cells. On the basis of findings, we suggest that acetylcholine induces activation of PDPK1 and enhances HIF-1α expression in pancreatic cancer cells under hypoxic conditions by reducing phosphorylation of YAP at serine 397.

### Acetylcholine acts via nAChR-α7 to enhance HIF-1α signaling, promoting pancreatic cancer growth under hypoxic conditions and *in vivo*

Next, to corroborate our *in vitro* data, we explored whether acetylcholine acts through the nAChR-α7 signaling pathway to contribute to the growth and survival of pancreatic cancer organoids under hypoxic conditions. To this end, organoids were treated with the nAChR-α7-specific inhibitor α-bungarotoxin (10 nM) or left untreated for 1 h, followed by treatment with or without 5 μM acetylcholine for 1 h and exposure to 20% or 5% O_2_ for 7 days.

**Figure 6:**
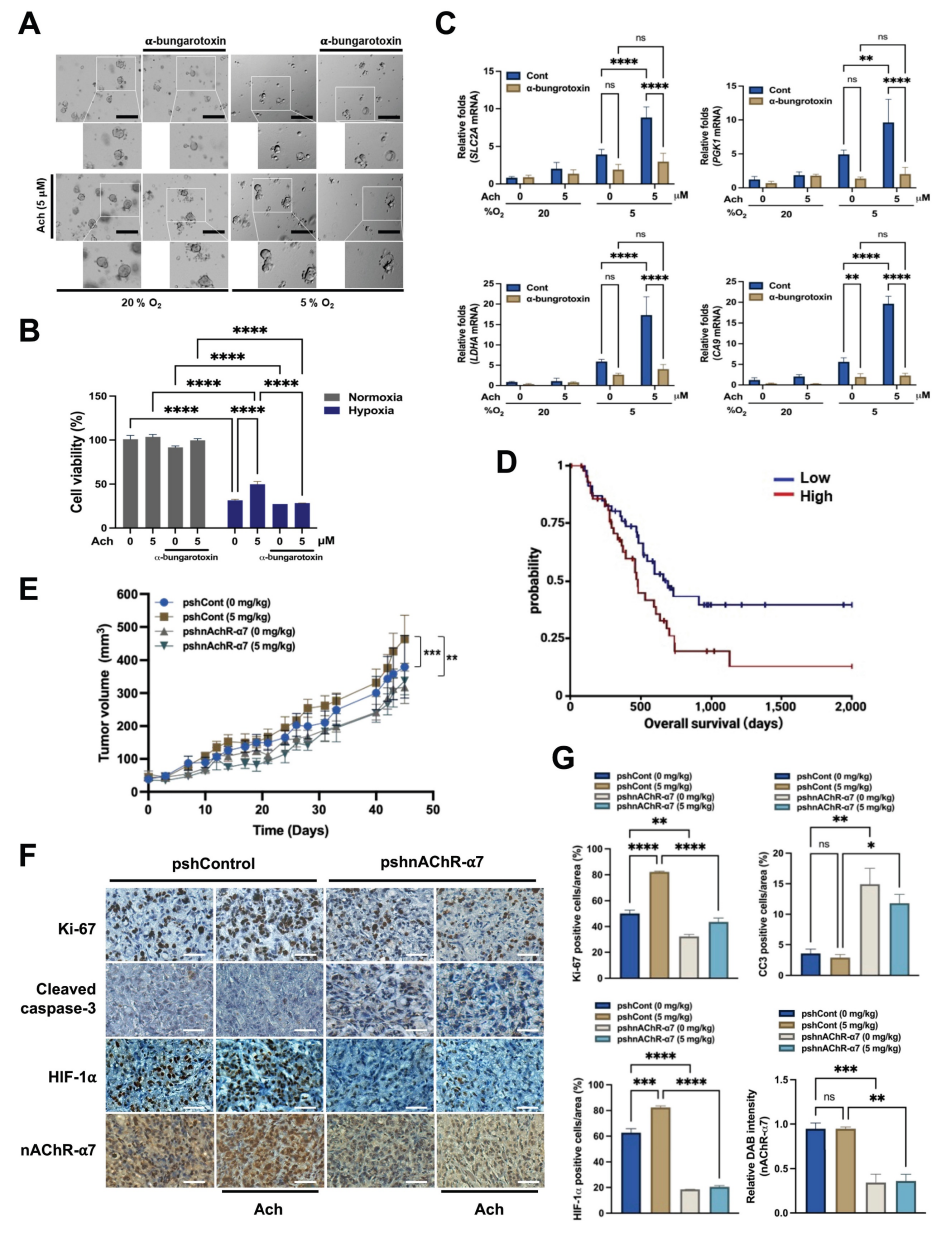
Acetylcholine enhances HIF-1α signaling via nAChR-α7, promoting pancreatic cancer growth in hypoxia and *in vivo*. **(A)** Representative bright-field images of pancreatic cancer organoids cultured for 7 d in organoid medium, with or without α-bungarotoxin or acetylcholine, under both normoxic and hypoxic conditions. Scale bars indicate 200 μm. **(B)** Assessment of the variability of pancreatic cancer organoids treated with or without α-bungarotoxin or acetylcholine and cultured in normoxic or hypoxic conditions for 2 d using the ATP-Glo assay. **(C)** Pancreatic cancer organoids were incubated with 10 nM α-bungarotoxin or 5 μM acetylcholine or left untreated for 1 h, followed by exposure to 20% or 5% O_2_ for 2 days before harvesting. HIF-1α target genes (*PGK1, LDHA, CA9* and* SLC2A*) were examined by qPCR using *18S rRNA* as an internal control. Data are presented as means ± SEM (*P < 0.05, **P < 0.01, ***P < 0.001, ****P < 0.0001; ANOVA). Ns indicates no significance. **(D)** Kaplan-Meier overall survival of TCGA-PAAD patients stratified by *CHRNA7* CNV (log-rank p = 0.03369; log-rank statistic = 4.499; n = 91). **(E)** Tumor models were generated by subcutaneously injecting AsPC-1/pshCont or AsPC-1/pshnAChR-α7 cells (2 × 10^6^ cells/mouse) into the right flank of mice. Mice were assigned to different groups (3-4 mice per group) and administered acetylcholine (5 mg/kg) by tail vein injection 2-times a week for 45 d. Tumor sizes were measured every 3 to 7 d using a digital caliper, and tumor volume was calculated using the following formula: TV = length × (width)^2^ × 0.5. Results are presented as means ± SEM (n = 3-4 per group, **P < 0.01, ***P < 0.001' ANOVA) **(F)** Immunohistochemical analyses of AsPC-1/pshCont and AsPC-1/pshnAChR-α7 xenograft tumors. Sections were stained for nAChR-α7, HIF-1α, proliferation (Ki-67) and apoptosis (CC3) using 3,3′-DAB. Scale bar, 100 μm. **(G)** Quantification of nAChR-α7, HIF-1α, proliferative marker Ki-67 and apoptotic marker CC3 in AsPC-1/pshCont and AsPC-1/pshnAChR-α7 xenograft tumors. Results are presented as means ± SEM (n = 3 per group) *P < 0.05, **P < 0.01, ***P < 0.001, ****P < 0.0001; ANOVA). Ns indicates no significance.

Under normoxic conditions, acetylcholine slightly enhanced the growth of pancreatic cancer organoids, whereas α-bungarotoxin exerted a minor inhibitory effect on their growth (**Figure 6A**). Conversely, under hypoxic conditions, acetylcholine restored the hypoxia-induced reduction in pancreatic cancer organoid growth, an effect that was inhibited by α-bungarotoxin (**Figure 6A**). Furthermore, acetylcholine promoted the viability of pancreatic cancer organoids in hypoxia; this too was prevented by α-bungarotoxin (**Figure 6B**). To investigate whether the ability of acetylcholine to counteract the hypoxia-induced reduction in pancreatic cancer organoid growth involves nAChR-α7-mediated enhancement of the HIF-1α signaling pathway, we first pretreated pancreatic cancer organoids with or without 10 nM α-bungarotoxin for 1 h, and then treated them with or without 5 μM acetylcholine for 1 h, followed by exposure to 20% or 5% O_2_. After 2 d of culture, pancreatic cancer organoids were harvested for evaluation of acetylcholine-induced activation of the HIF signaling pathway. Our qPCR findings demonstrated that acetylcholine enhanced the expression of HIF-1α target genes in pancreatic cancer organoids under hypoxic conditions (**Figure 6C**). Furthermore, as illustrated in **Figure 6C**, application of α-bungarotoxin significantly decreased acetylcholine-induced enhancement of HIF-1α target gene expression in these organoids in a hypoxic setting.

For clinical relevance, we analyzed TCGA-PAAD patient survival based on *CHRNA7* CNV data from the UCSC XenaBrowser. Overall survival of patients with *CHRNA7* amplification was significantly shorter compared with that of patients without *CHRNA7* amplification (log-rank p = 0.03392; log-rank test statistics = 4.499; n = 91) (**Figure 6D**). Stratification according to *CHRNA7* mRNA expression level revealed a non-significant trend toward shorter survival in high-expressing patients (log-rank p = 0.7022) (**Supplementary Figure S9**). These findings suggest that CNV is a more robust predictor of prognosis than mRNA level alone. Overall, acetylcholine enhanced HIF-1α signaling through modulation of the nAChR-α7 signaling pathway, promoting pancreatic cancer organoid growth under hypoxic conditions; moreover, *CHRNA7* CNV amplification was correlated with reduced patient survival.

Finally, to confirm the physiological relevance of these observations, we established an *in vivo* xenograft model using AsPC-1 cells stably transfected with pshCont or pshnAChR-α7. Female BALB/c nude mice were subcutaneously implanted with these cells and treated with or without acetylcholine (5 mg/kg). As shown in **Figure 6E**, acetylcholine promoted the growth of pshCont-transfected xenografts, a stimulatory effect that was absent in pshnAChR-α7-transfected tumors. Additionally, nAChR-α7 knockdown substantially delayed tumor growth compared with controls (**Figure 6E**). Immunohistochemical analyses demonstrated that acetylcholine treatment enhanced HIF-1α expression in control xenografts, whereas nAChR-α7 knockdown led to reduced HIF-1α expression, regardless of acetylcholine administration. This decrease was accompanied by reduced proliferation (Ki67) and enhanced apoptosis (cleaved cacspase-3 [CC3]) (**Figure 6F, G**). Together, these findings indicate that acetylcholine facilitates pancreatic tumor growth by enhancing HIF-1α signaling through nAChR-α7, and that inhibition of nAChR-α7 disrupts this signaling axis, leading to impaired tumor proliferation and increased apoptotic cancer cell death.

## Discussion

In the present work, we identified a mechanism by which acetylcholine enhances the expression of HIF-1α in pancreatic cancer cells under conditions of hypoxia. Mechanistic analyses suggest that acetylcholine activates PDPK1, thereby downregulating phosphorylation of YAP at serine 397 and promoting its translocation from the cytosol to the nucleus, where it enhances HIF-1α production.

The peripheral autonomic nervous system is reported to regulate gene expression in primary tumors and their surrounding milieu 1-3. Autonomic nervous system-mediated expression of genes in primary solid tumors can promote metastasis through stimulation of macrophage infiltration, EMT, angiogenesis, inflammation and tumor invasion, as well as through inhibition of cellular immune responses and programmed cell death 1. The autonomic nervous system is divided into sympathetic and parasympathetic branches, which exert variable effects on cancers according to type and stage 2. The exocrine pancreas is densely innervated by both parasympathetic and sympathetic nervous systems 4. Recent reports have demonstrated that the mechanisms by which the pancreatic autonomic nervous system influences tumor development are more complex for PDAC than for other cancers 2. Previous studies have described an antitumor effect of pancreatic parasympathetic cholinergic signaling in PDAC 5,6 and conversely demonstrated involvement of the sympathetic nervous system in tumor formation 7. In view of the contribution of the autonomic nervous system to malignancy, there has been a substantial increase in research interest in the effects of various neurotransmitters on carcinogenesis 8. The peripheral autonomic neurotransmitters epinephrine, norepinephrine, dopamine, serotonin, and acetylcholine have been shown to play roles in the malignant transformation of various cancer types 9.

Nicotine-induced YAP and HIF-1α expression is reported to induce EMT in PDAC during hypoxia 21. Moreover, nicotine binds nAChR and stimulates the proliferation of small-cell lung cancer cells 15. Acetylcholine is a neurotransmitter of the parasympathetic system whose biological activity is mediated by nAChRs and mAChRs in both the central and peripheral nervous systems 14. Poor vascular structure within solid tumors causes hypoxia, which is a particularly important feature in the microenvironment of PDAC 21. The hypoxic condition applied in our cell culture experiments (0.5% O_2_, corresponding to ~3.8 mmHg pO_2_) aligns with values reported in clinical studies of PDAC tumors. Given that intratumoral O_2_ levels in human PDAC range from 0 to 5.3 mmHg 46, our experimental setup closely mimics the hypoxic microenvironment encountered *in vivo*. The hypoxic environment triggers HIF-1α signaling associated with the expression of a number of genes involved in angiogenesis, tumor growth, metabolic reprogramming, metastasis, chemoresistance, and radioresistance in cancer cells 25. Accordingly, to ascertain whether acetylcholine secreted by the autonomic nervous system affects the HIF signaling pathway of pancreatic cancer cells under hypoxic conditions, we conducted experiments focusing on an environment in which the autonomic nervous system is densely distributed. Acetylcholine enhanced the viability of pancreatic cancer organoids in hypoxia (**Figure 6A**) and potentiated hypoxia-induced expression of HIF-1α and its target genes in pancreatic cancer cells (**Figure 1**, **Supplementary Figure S1**). As noted above, nicotine induces YAP and HIF-1α expression via nAChRs, in particular nAChR-α7, in PDAC 21. Accordingly, we additionally investigated the identity of AChRs involved in acetylcholine-induced enhancement of HIF-1α signaling in pancreatic cancer cells in a hypoxic environment. Notably, we found that nAChR-α7 was expressed in all pancreatic cancer cell lines used in this study (**Figure 2A, B**) and was involved in acetylcholine-induced enhancement of the HIF-1α signaling pathway in hypoxic pancreatic cancer cells (**Figure 2C-H**). Experiments with the mAChR-specific inhibitor, scopolamine, and nAChR-α7-specific inhibitor, α-bungarotoxin, confirmed a major contribution of nAChR-α7 to acetylcholine-induced enhancement of HIF-1α expression under conditions of hypoxia in the cell lines examined (**Supplementary Figure S3-S4**).

Based on previous reports on nAChR-α7-mediated YAP and HIF-1α expression in PDAC 21, we further explored the mechanisms by which acetylcholine regulates HIF-1α in hypoxic pancreatic cancer cells. In the presence of CHX, a blocker of *de novo* protein synthesis, acetylcholine increased HIF-1α protein stability (**Figure 3C, D, Supplementary Figure S5D, E**). Nicotine-induced YAP stimulates an increase in the expression of HIF-1α, which further enhances YAP expression 21. Knockdown of nAChR-α7 inhibited acetylcholine-induced enhancement of HIF-1α but not YAP expression (**Figure 4A, B**). siRNA experiments designed to ascertain the signaling molecules upstream of HIF-1α and YAP revealed that, in hypoxic pancreatic cancer cells, siYAP significantly inhibited both HIF-1α and YAP (**Figure. 4C, D**), whereas siHIF-1α exerted a significant inhibitory effect on HIF-1α, but not YAP (**Figure 4E, F**). Based on these results, we tested the hypothesis that acetylcholine promotes the translocation of YAP from the cytosol to the nucleus in a hypoxic environment, thereby enhancing expression of HIF-1α. As shown in **Figure 4G, H**, acetylcholine enhanced the translocation of cytoplasmic YAP and HIF-1α to the nucleus in hypoxic pancreatic cancer cells. In addition, siYAP significantly suppressed the acetylcholine-induced enhancement of HIF-1α target gene expression in these cells (**Figure 4J-M**). In hypoxic cancer cells, nuclear YAP is reported to increase HIF-1α protein stability 21. Consistent with previous reports, we found that YAP co-localizes with and binds to HIF-1α in nuclei of pancreatic cancer cells under hypoxic conditions (**Figure 4I**). Overall, our data support the theory that acetylcholine induces translocation of YAP from the cytosol to the nucleus, thereby enhancing HIF-1α expression in hypoxic pancreatic cancer cells.

**Figure 7:**
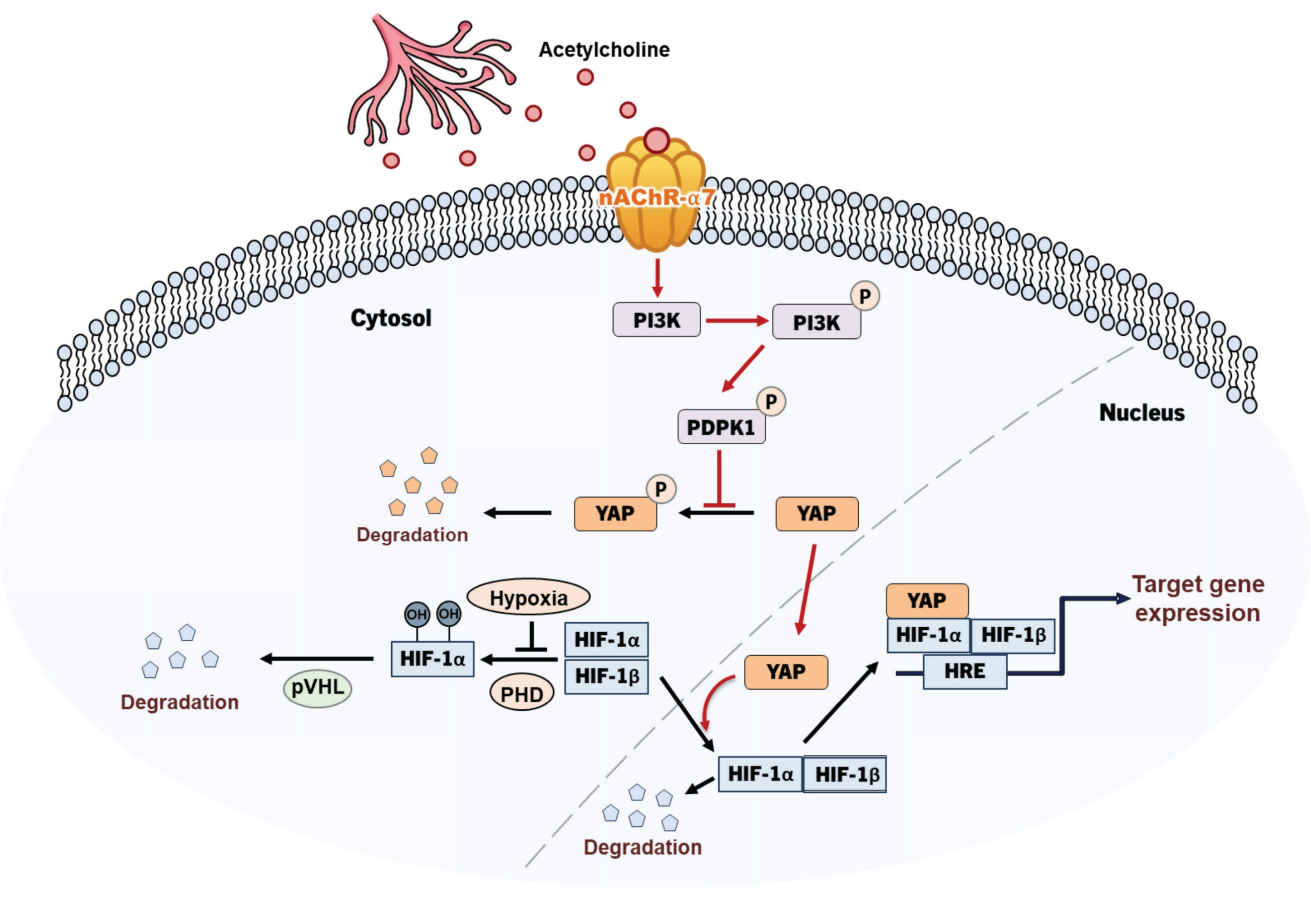
Schematic illustration of acetylcholine-induced enhancement of HIF-1α expression through activation of the nAChR-α7/PI3K/PDPK1/YAP signaling pathway in hypoxic pancreatic cancer cells.

To clarify the signaling pathways involved in the acetylcholine-induced enhancement of HIF-1α expression, we conducted studies focusing on how acetylcholine promotes the translocation of YAP in pancreatic cancer cells in hypoxia. Previous studies suggested that YAP is regulated through reduced serine 397 phosphorylation, which promotes its migration from the cytosol to the nucleus 34. According to a separate study 36, PI3K-activated PDPK1 induces translocation of cytosolic YAP to the nucleus by reducing its phosphorylation. Abnormal activation of PI3K signaling is common in cancer and acts to regulate tumor cell survival, growth, proliferation and migration 36. In pancreatic cancer, mutant KRAS induces PI3K signaling 42, and nicotine-induced activation of nAChR-α7 stimulates the PI3K signaling pathway through Src 45. Data from the current study showed that acetylcholine enhances hypoxia-induced phosphorylation of PDPK1 in pancreatic cancer cells (**Figure 5A, B**). In support of this, siPDPK1 effectively suppressed the expression of YAP and HIF-1α (**Figure 5A, B**). However, neither siYAP nor siHIF-1α inhibited activation or expression of PDPK1 in pancreatic cancer cells (**Figure 5C-F**). PI3K-mediated PDPK1 activation reduces serine 397 phosphorylation of intracellular YAP 36. Consistent with this, we found that acetylcholine-induced enhancement of PDPK1 activation led to reduced phosphorylation of cytosolic YAP in pancreatic cancer cells, an effect that was reversed by siPDPK1 (**Figure 5G, H**). In view of these results, we investigated whether acetylcholine induces nAChR-α7-mediated enhancement of HIF-1α expression through activation of PI3K, showing that nAChR-α7 knockdown (pshnAChR-α7) significantly inhibited acetylcholine-induced enhancement of PI3K activation in hypoxic pancreatic cancer cells, as illustrated in **Figure 7**. Together, the findings suggest that, under hypoxic conditions, acetylcholine stimulates the YAP/HIF-1α signaling pathway through activation of nAChR-α7/PI3K/PDPK1 signaling in pancreatic cancer cells.

To establish the clinical relevance of nAChR-α7, we analyzed TCGA-PAAD data. Patients with *CHRNA7* copy number amplification exhibited significantly poor overall survival compared with patients without amplification (**Figure 6D**). In contrast, stratification according to *CHRNA7* mRNA expression revealed a non-significant trend (p = 0.7022) (**Supplementary Figure S9**). These results suggest that copy number alterations may provide a more robust prognostic indicator than mRNA expression alone. Moreover, the nAChR-α7-specific inhibitor, α-bungarotoxin, reduced the viability of pancreatic cancer organoids under hypoxic conditions (**Figure 6A, B**). Consistent with our findings, acetylcholine enhanced pancreatic tumor growth *in vivo* through nAChR-α7-mediated activation of HIF-1α signaling. Knockdown of nAChR-α7 abrogated this effect, highlighting the critical role of this receptor in acetylcholine-driven tumor progression in hypoxia. On the basis of these findings, we conclude that acetylcholine enhances HIF-1α expression through regulation of the nAChR-α7/PI3K/PDPK1/YAP signaling pathway in pancreatic cancer cells under hypoxic conditions (**Figure 7**). Overall, our results support HIF-1α as a promising therapeutic target for acetylcholine-associated pancreatic cancer.

## Supplementary Material

Supplementary figures.
