# Supplementary material for: Acetylcholine enhances HIF-1α signaling in pancreatic cancer cells under hypoxia through the nAChR-α7/PDPK1/YAP pathway
Source: Int J Biol Sci. 2026 Jan 8;22(3):1369–88. doi: 10.7150/ijbs.117013 (PMC12837655; doi:10.7150/ijbs.117013)

Acetylcholine enhances HIF-1α signaling in pancreatic cancer cells under hypoxia through the nAChR-α7/PDPK1/YAP pathway

Cho et al.,

Supplementary Figures

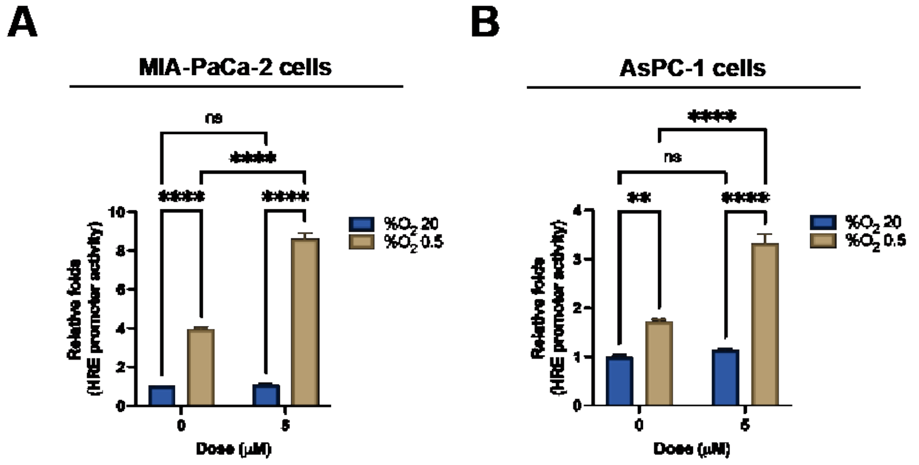

Supplementary Figure S1. Acetylcholine enhances the expression of HIF-1α target genes in pancreatic cancer cells under hypoxia. **(A-B)** MIA-PaCa-2 **(A)** and AsPC-1 **(B)** cells were co-transfected with p3×HRE-*luc* and pRL-*luc* and incubated with or without 5 μM acetylcholine for 1 h. Cells were subsequently exposed to 20% or 5% O_2 for 24 h. Luciferase activity in whole-cell lysates was normalized to that of *Renilla* luciferase. Data are presented as means ± SEM (***P* < 0.01, *****P* < 0.0001; ANOVA). Ns indicates no significance.

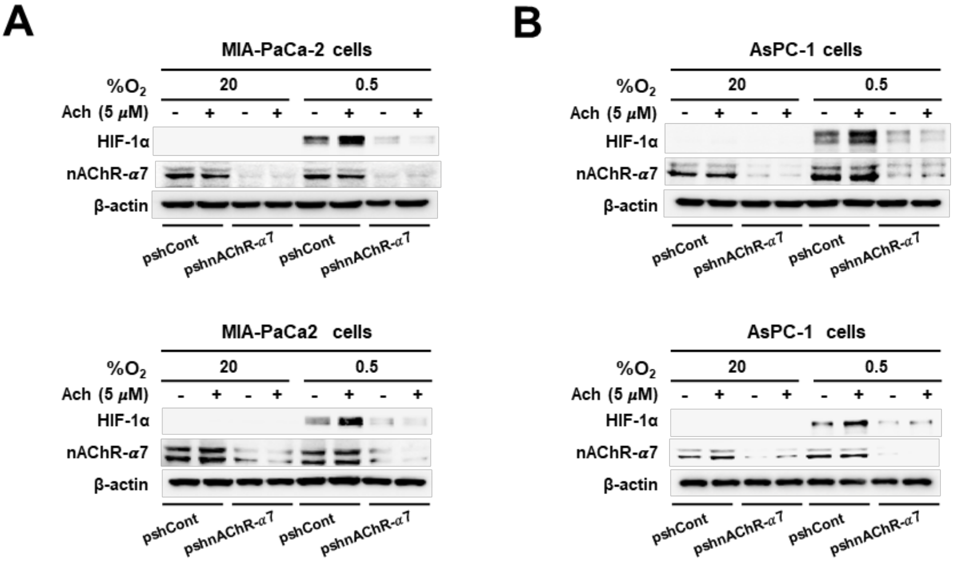

Supplementary Figure S2. Acetylcholine enhances HIF-1α expression via nAChR-α7 in pancreatic cancer cells under hypoxia. **(A-B)** pshCont- or pshnAChR-α7-transfected MIA-PaCa-2 **(A)** and AsPC-1 **(B)** cells were treated with 5 μM acetylcholine or left untreated for 1 h, exposed to 20% or 0.5% O_2 for 24 h, and harvested. The indicated proteins in whole-cell lysates were analyzed by immunoblotting.

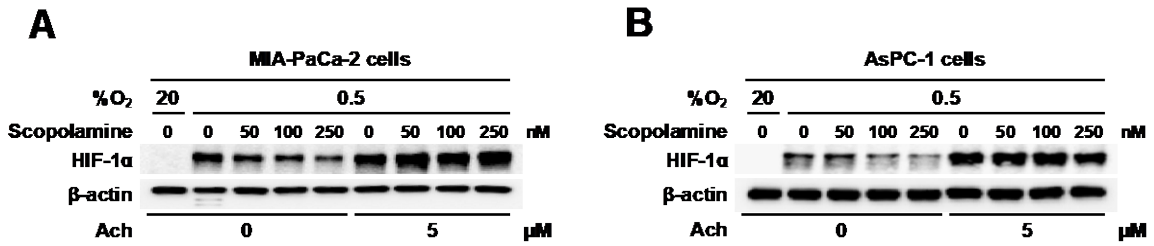

Supplementary Figure S3. Effects of scopolamine on acetylcholine-mediated enhancement of HIF-1α expression in pancreatic cancer cells under hypoxia. **(A-B)** MIA-PaCa-2 **(A)** and AsPC-1 **(B)** cells were treated with a range of concentrations of scopolamine (0–250 nM) for 1 h, followed by 5 μM acetylcholine for 1 h, and exposed to 20% or 0.5% O_2. After a 24 h incubation period, cells were harvested and whole-cell lysates analyzed via immunoblotting for the indicated proteins.

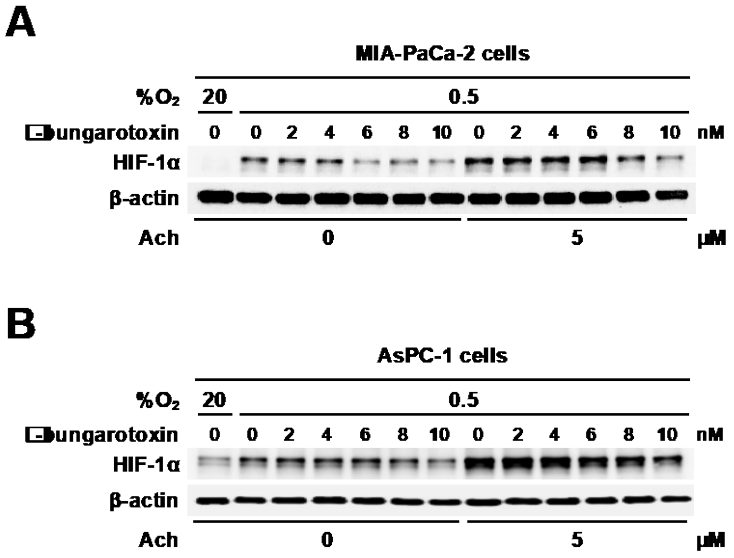

Supplementary Figure S4. Effect of α-bungarotoxin on acetylcholine-mediated enhancement of HIF-1α expression in pancreatic cancer cells under hypoxia. **(A-B)** MIA-PaCa-2 **(A)** and AsPC-1 **(B)** cells were treated with different concentrations of α-bungarotoxin (0–10 μM) for 1 h, followed by 5 μM acetylcholine for 1 h, and exposed to 20% or 0.5% O_2. After a 24 h incubation period, cells were harvested, and the indicated proteins analyzed via immunoblotting in whole-cell lysates.

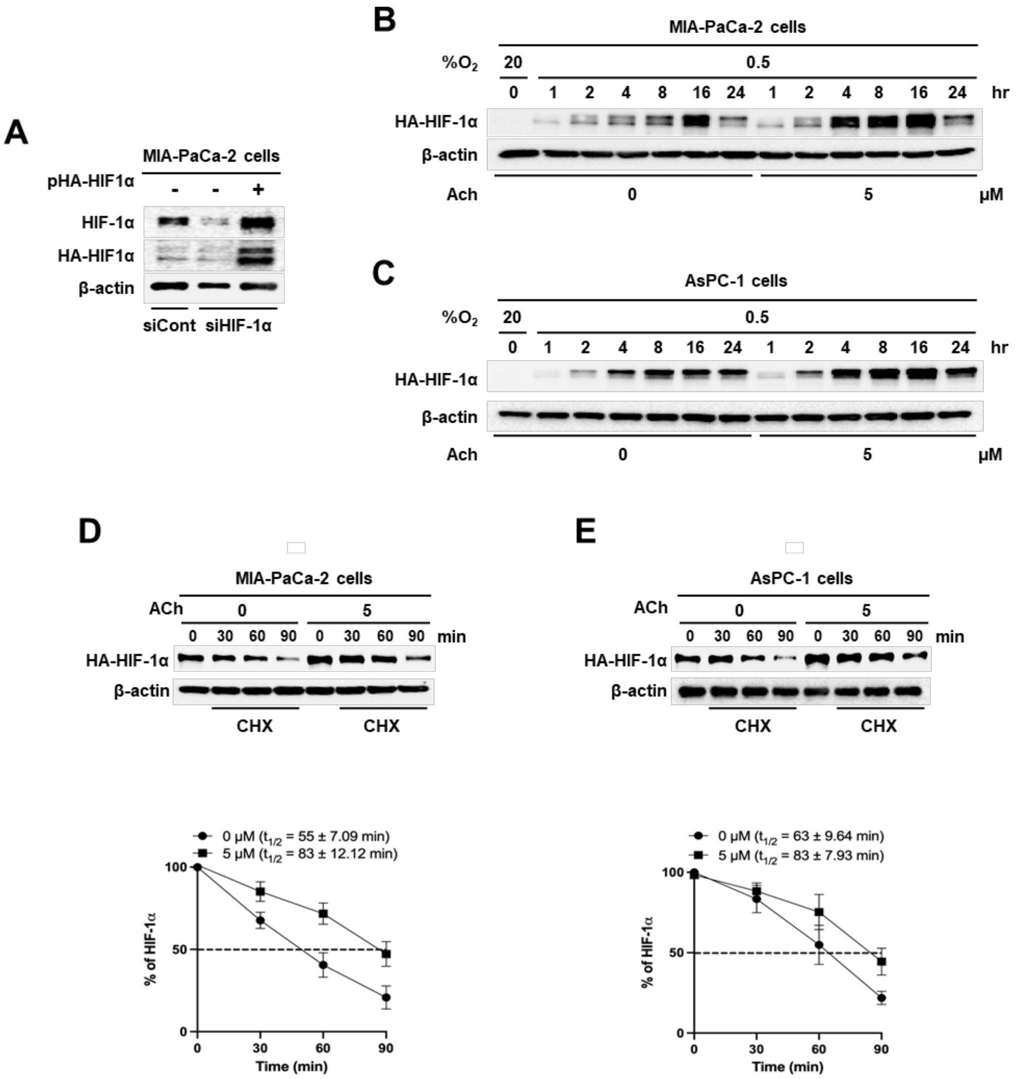

Supplementary Figure S5. Acetylcholine increases HIF-1α protein stability in pancreatic cancer cells under hypoxia**. (A)** MIA-PaCa-2 cells were transfected with siCont, siHIF-1α, or siHIF-1α along with pHA-HIF-1α for 24 h and subsequently exposed to 0.5% O_2. After a 24 h incubation, cells were harvested. HIF-1α protein levels were examined via immunoblot analysis using β-actin as an internal control. **(B-C)** MIA-PaCa-2 **(B)** and AsPC-1 **(C)** cells were transfected with siHIF-1α and pHA-HIF-1α for 24 h, treated with or without 5 μM acetylcholine, and exposed to normoxia (20% O2) or hypoxia (0.5% O_2) for the indicated times. Following harvest of cells at the indicated times, the HA-HIF-1α expression and β-actin was analyzed using immunoblot analysis. **(D-E)** MIA-PaCa-2 **(D)** and AsPC-1 **(E)** cells were transfected with siHIF-1α and pHA-HIF-1α for 24 h, treated with or without 5 μM acetylcholine for 1 h, and exposed to hypoxia (0.5% O_2) for 16 h. Then the cells were treated with 10 μg/mL CHX or left untreated and harvested at the indicated times. HIF-1α protein levels were examined via immunoblot analysis using β-actin as an internal control. Upper panel: Representative images of immunoblot analysis. Lower panel: HIF-1α protein levels were quantified using Image J and band intensities normalized to that of β-actin (the band intensity at t_0 is defined as 100%).

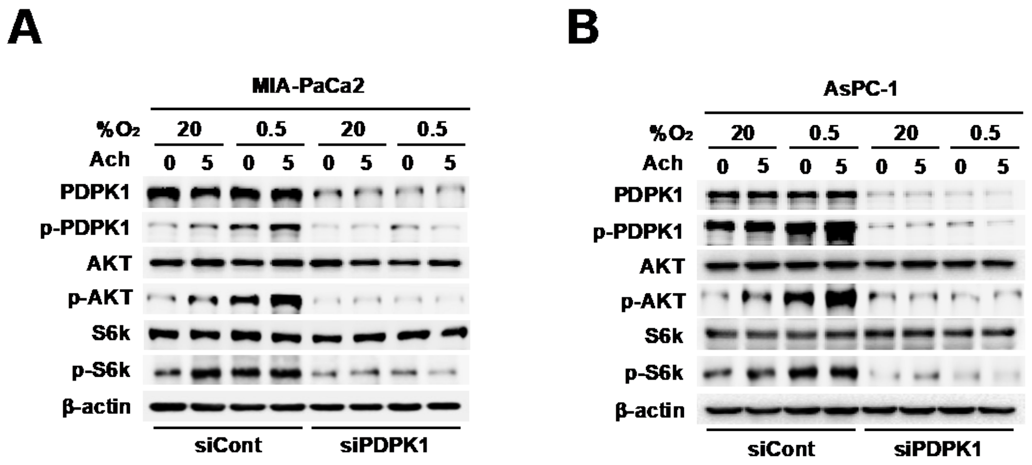

Supplementary Figure S6. Acetylcholine enhances activation of PDPK1 and its target proteins in pancreatic cancer cells under hypoxia. **(A-B)** siCont- or siPDPK1-transfected MIA-PaCa-2 **(A)** and AsPC-1 **(B)** cells were treated with or without 5 μM acetylcholine for 1 h and exposed to 20% or 0.5% O_2. After a 24 h incubation period, cells were harvested, and the indicated proteins analyzed in whole-cell lysates via immunoblotting.

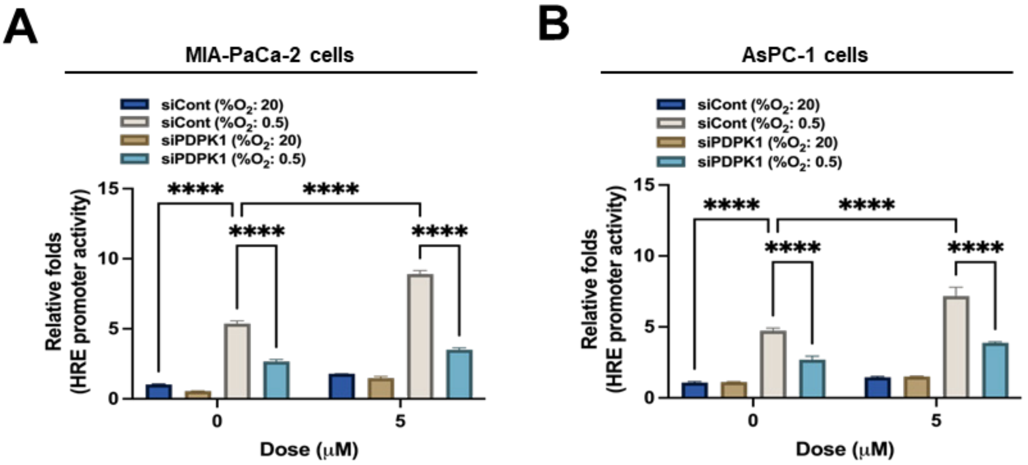

Supplementary Figure S7. Acetylcholine enhances HIF-1α signaling pathway through regulating PDPK1 activation in pancreatic cancer cells under hypoxia. **(A-B)** siCont- or siPDPK1-transfected MIA-PaCa-2 **(A)** and AsPC-1 **(B)** cells were co-transfected with p3×HRE-*luc* and pRL-*luc*, followed by treatment with or without 5 μM acetylcholine for 1 h. Cells were subsequently exposed to 20% or 5% O_2 for 24 h. Luciferase activity measured in whole-cell lysates was normalized to that of *Renilla* luciferase. Data are presented as means ± SEM (*****P* < 0.0001; ANOVA).

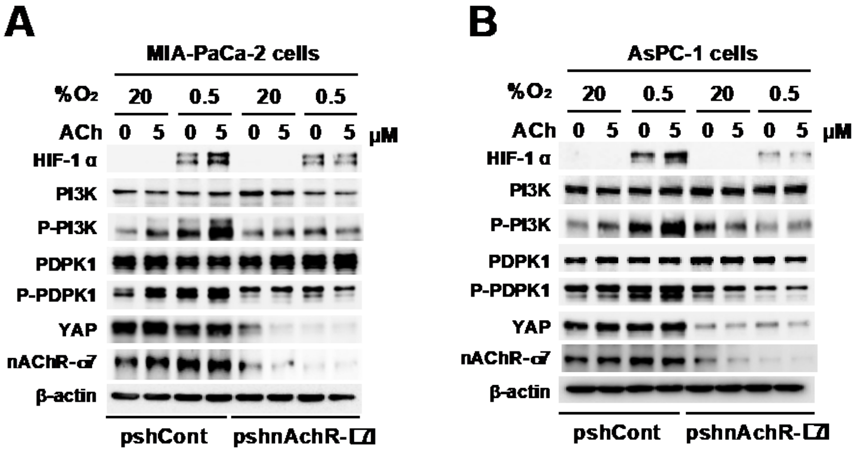

Supplementary Figure S8. Acetylcholine enhances HIF-1α signaling pathway through regulating nAChR-α7/PI3K/PDPK1/YAP signaling pathway in pancreatic cancer cells under hypoxia. **(A-B)** pshCont- or pshAChR-α7-transfected MIA-PaCa-2 **(A)** and AsPC-1 **(B)** cells were treated with or without 5 μM acetylcholine for 1 h and exposed to 20% or 0.5% O_2. After a 24 h incubation period, cells were harvested and the indicated proteins analyzed in whole-cell lysates via immunoblotting.

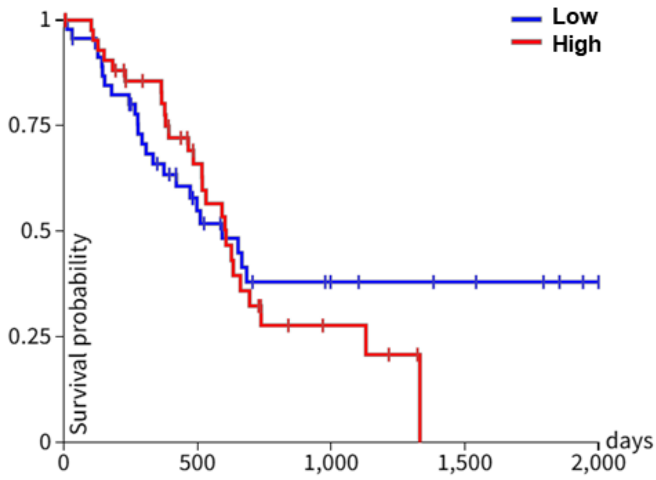

Supplementary Figure S9. Kaplan-Meier overall survival of TCGA-PAAD patients stratified by CHRNA7 mRNA expression (log-rank p = 0.7022; n = 91).

Supplementary Figure S10.

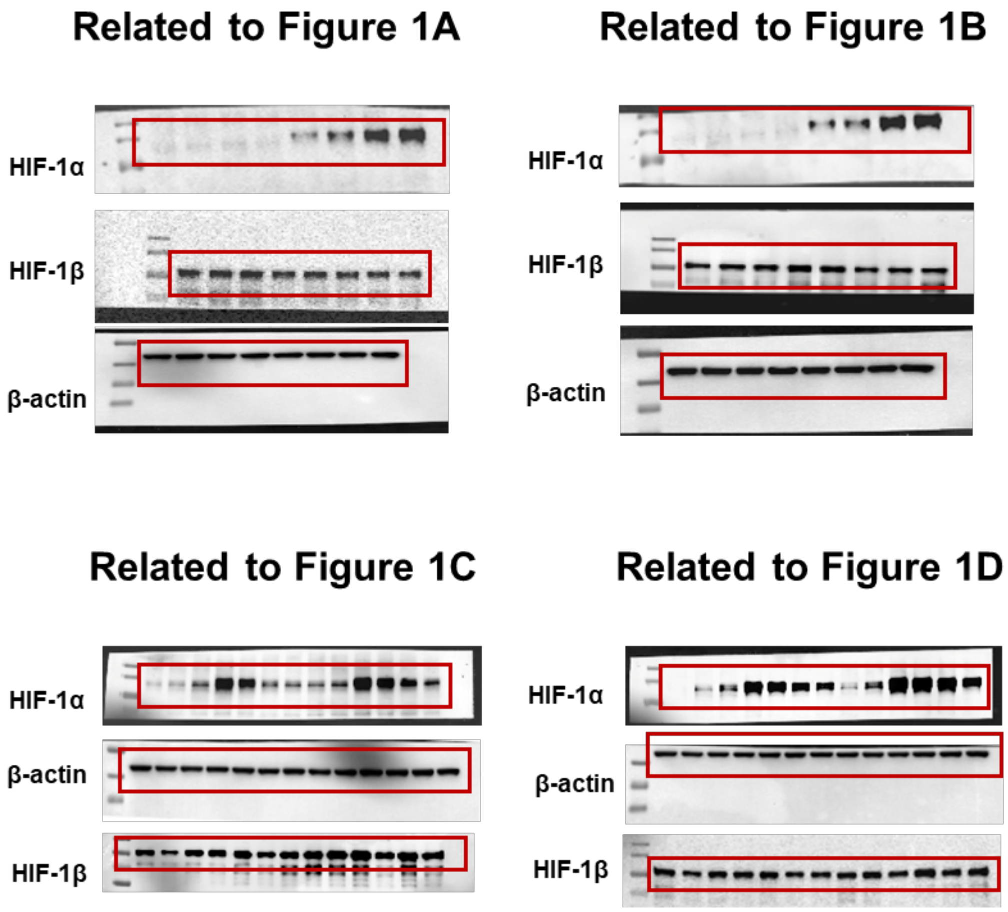

Related to Figure 2A

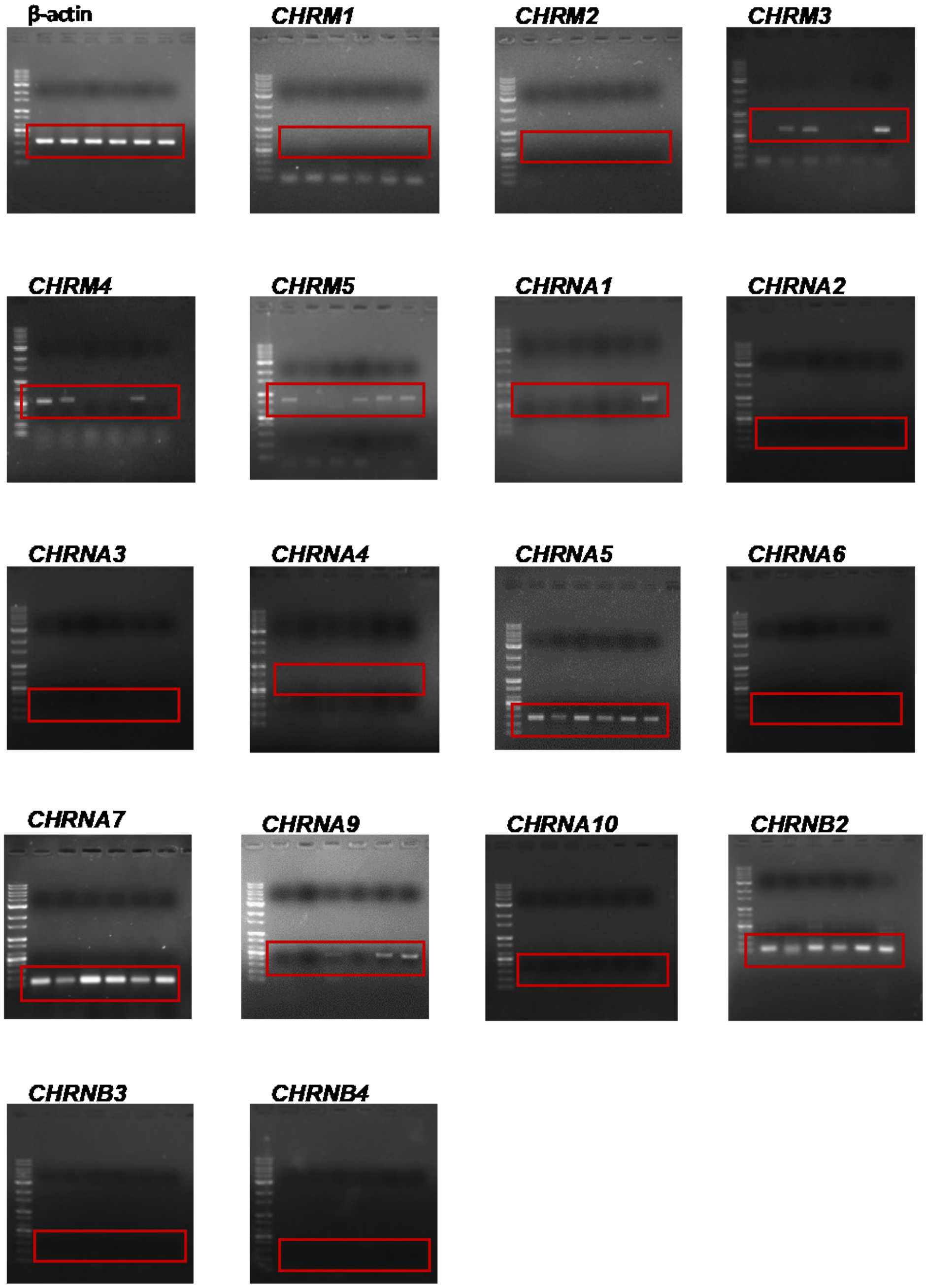

Related to Figure 2B

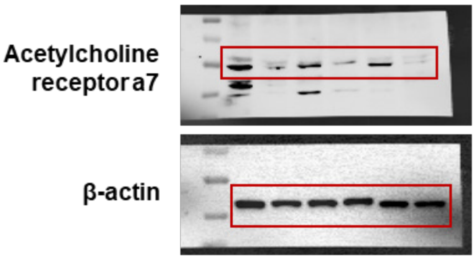

Related to Figure 2C

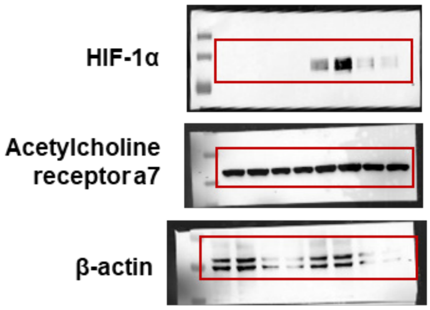

Related to Figure 2D

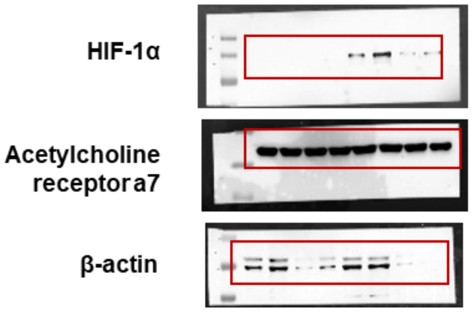

Related to Figure 3E

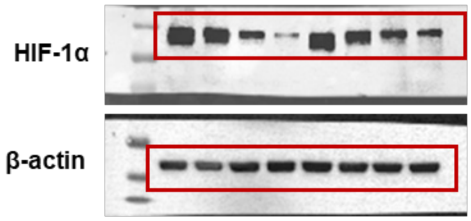

Related to Figure 3F

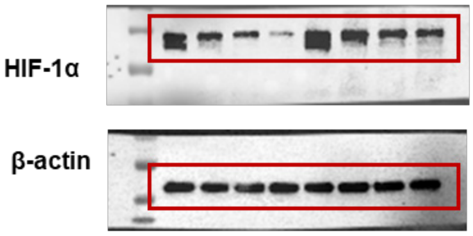

Related to Figure 3G

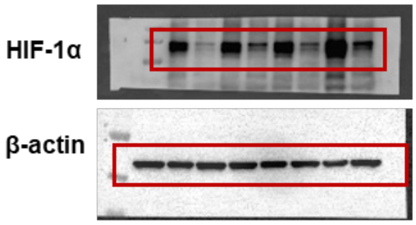

Related to Figure 3H

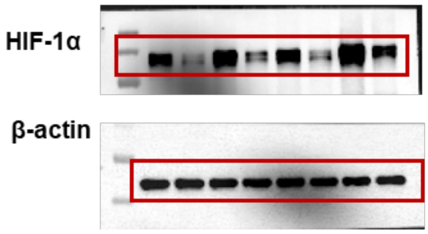

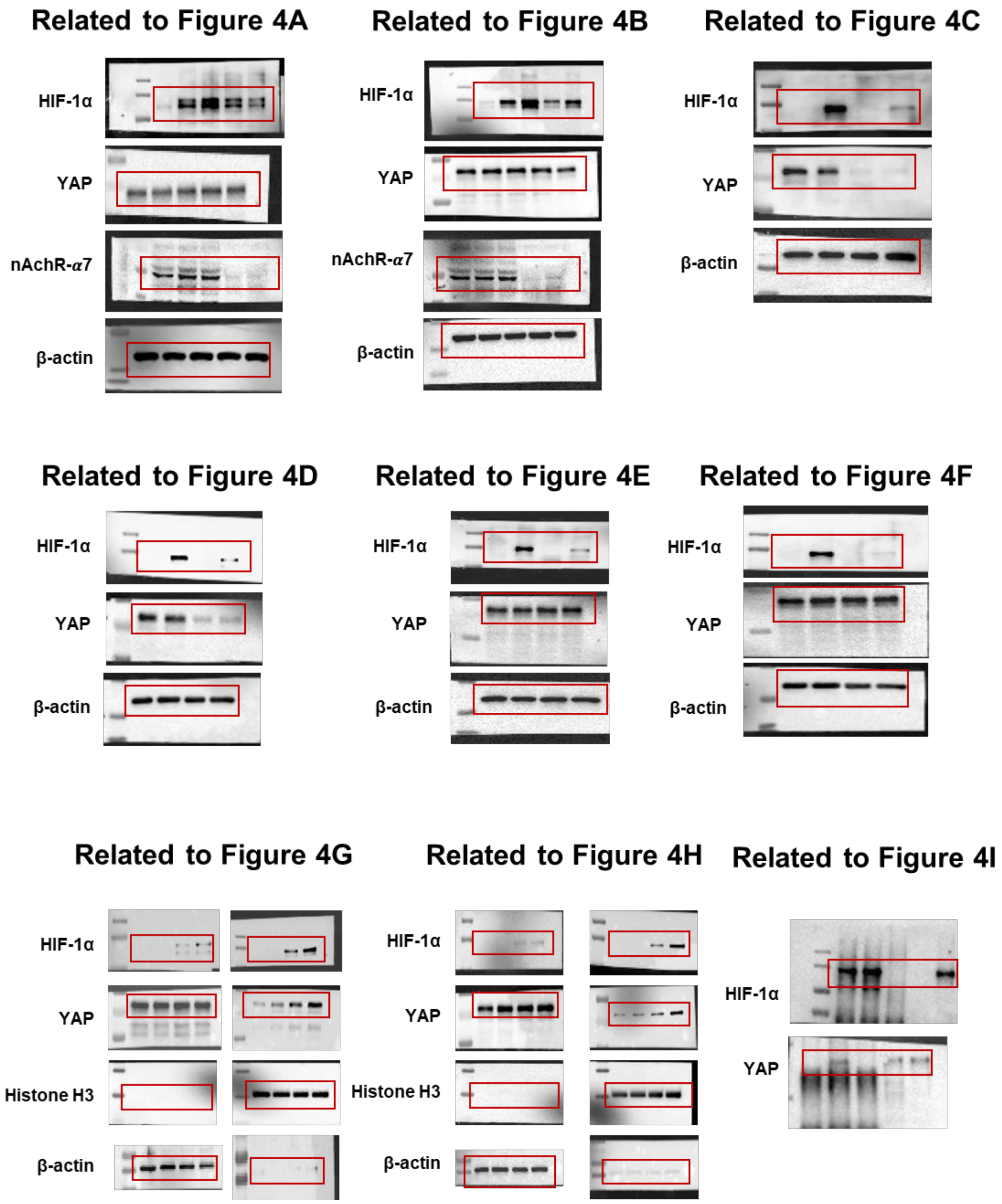

Related to Figure 4A
HIF-1α
YAP
nAchR-α7
β-actin
Related to Figure 4B
HIF-1α
YAP
nAchR-α7
β-actin
Related to Figure 4C
HIF-1α
YAP
β-actin
Related to Figure 4D
HIF-1α
YAP
β-actin
Related to Figure 4E
HIF-1α
YAP
β-actin
Related to Figure 4F
HIF-1α
YAP
β-actin
Related to Figure 4G
HIF-1α
YAP
Histone H3
β-actin
Related to Figure 4H
HIF-1α
YAP
Histone H3
β-actin
Related to Figure 4I
HIF-1α
YAP

Related to Figure 5A

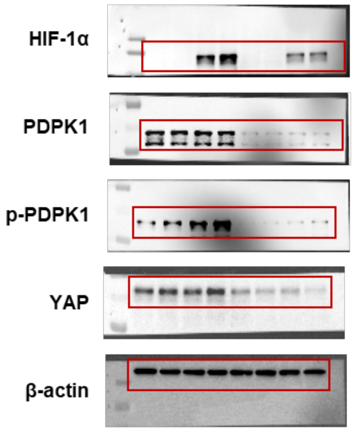

Related to Figure 5B

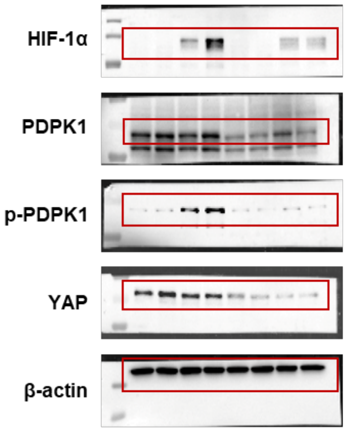

Related to Figure 5E

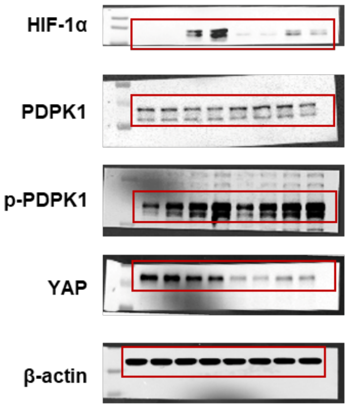

Related to Figure 5C

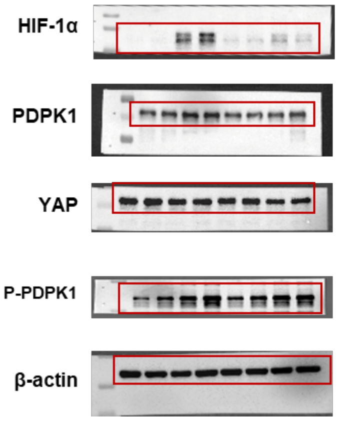

Related to Figure 5D

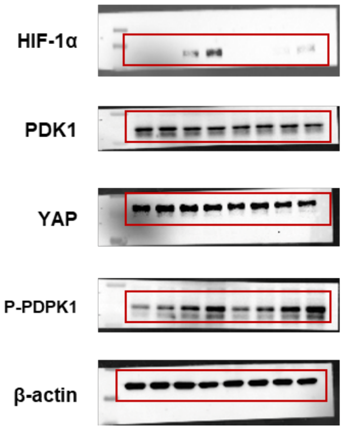

Related to Figure 5F

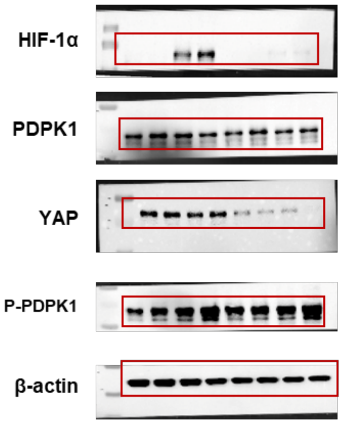

Related to Figure 5G

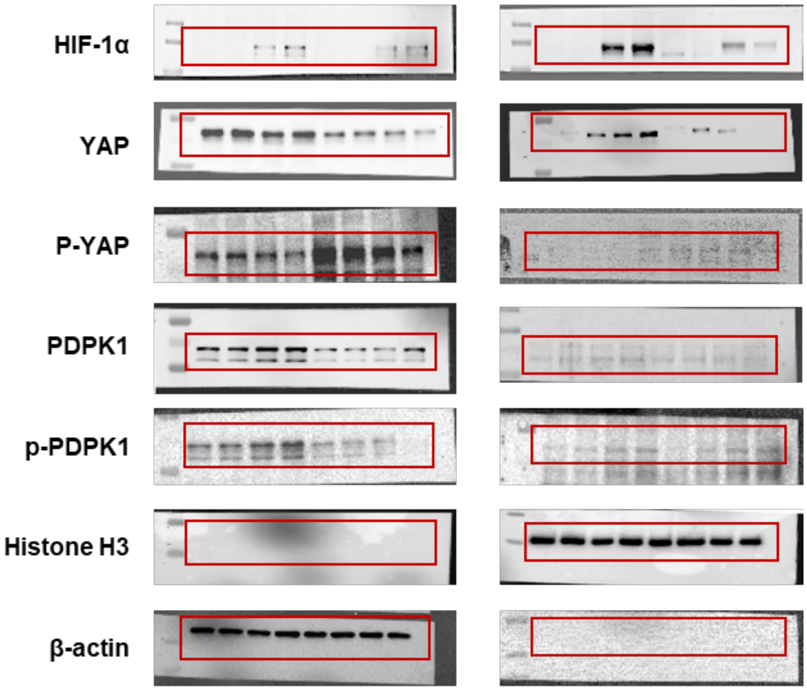

Related to Figure 5H

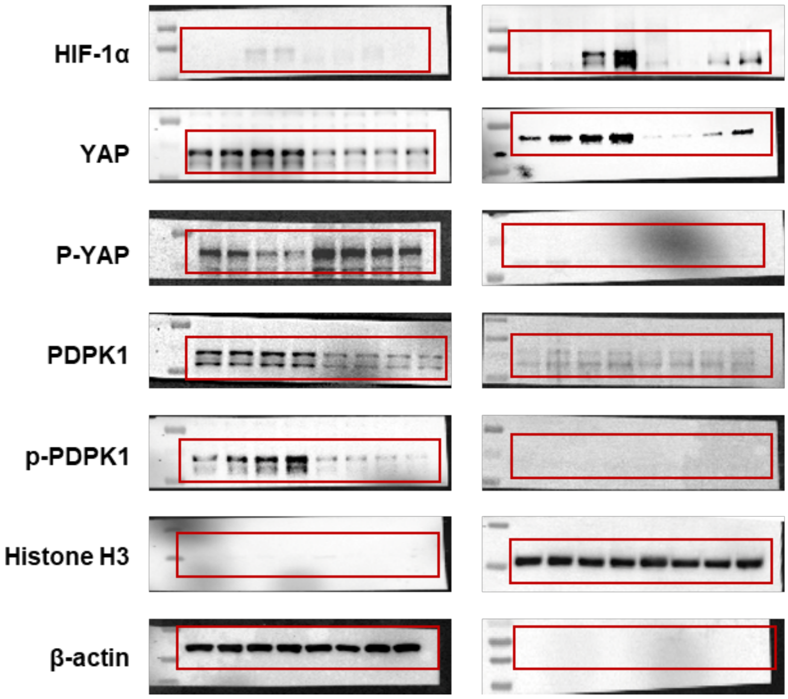

Related to Supplementary Figure S2A

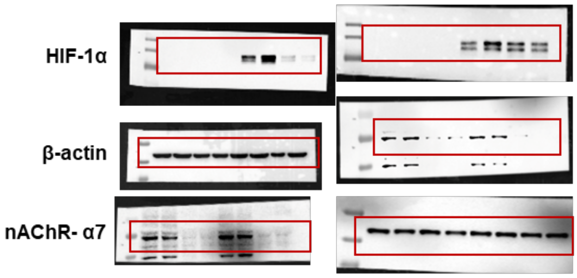

Related to Supplementary Figure S2B

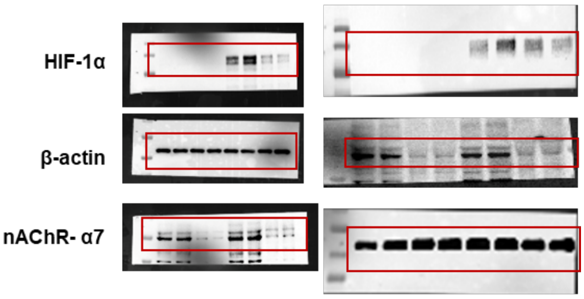

Related to Supplementary Figure S3A-B

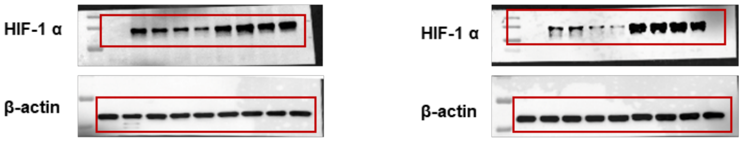

Related to Supplementary Figure S4A-B

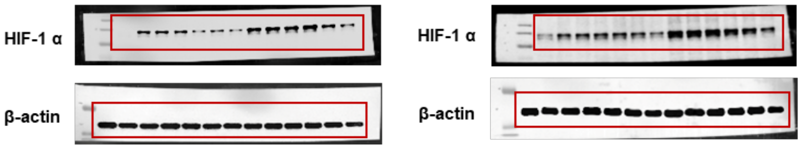

Related to Supplementary Figure S5A

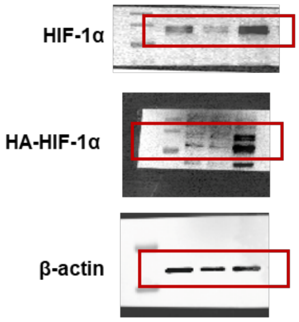

Related to Supplementary Figure S5B-C

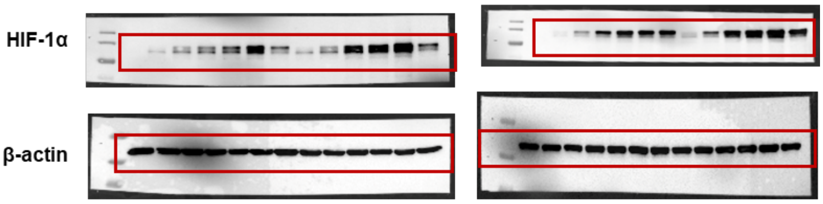

Related to Supplementary Figure S5D-E

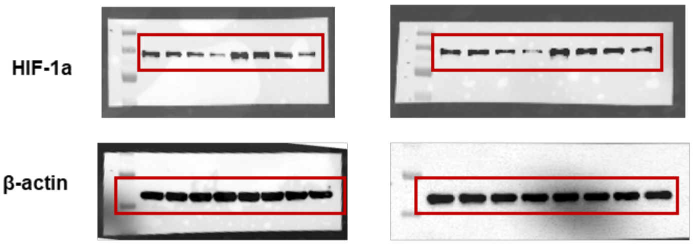

Related to Supplementary Figure S6A

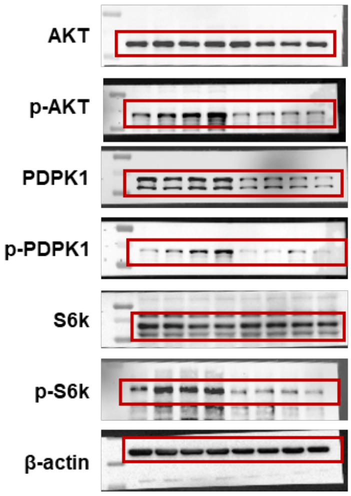

Related to Supplementary Figure S6B

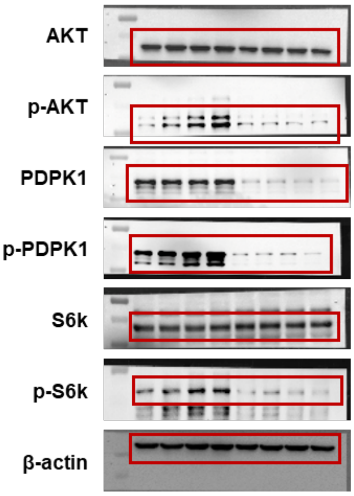

Related to Supplementary Figure S8A

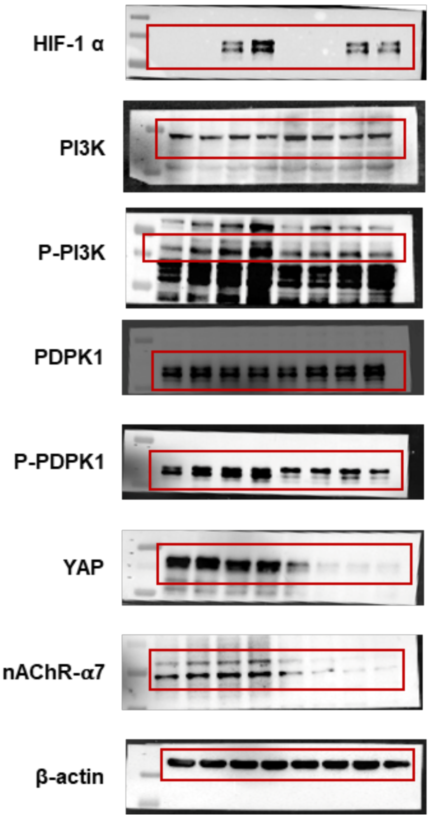

Related to Supplementary Figure S8B

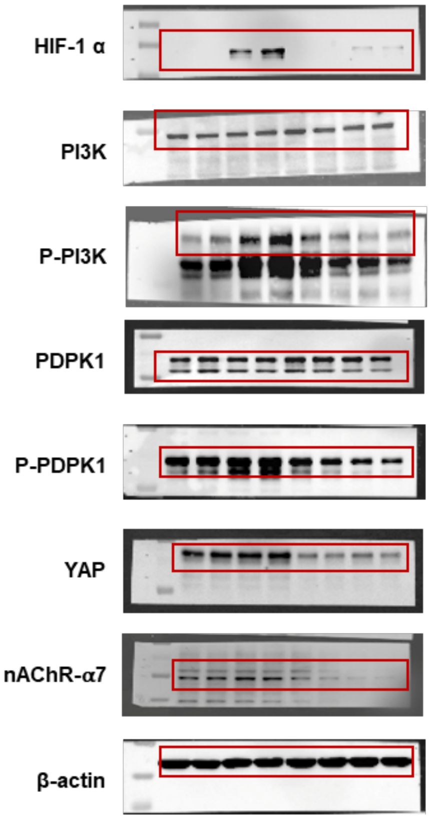

Supplement: Supplementary file 1 — Supplementary figures. [file ijbsv22p1369s1.pdf]
